# In vivo induction of activin A-producing alveolar macrophages supports the progression of lung cell carcinoma

**DOI:** 10.1038/s41467-022-35701-8

**Published:** 2023-01-17

**Authors:** Seiji Taniguchi, Takahiro Matsui, Kenji Kimura, Soichiro Funaki, Yu Miyamoto, Yutaka Uchida, Takao Sudo, Junichi Kikuta, Tetsuya Hara, Daisuke Motooka, Yu-Chen Liu, Daisuke Okuzaki, Eiichi Morii, Noriaki Emoto, Yasushi Shintani, Masaru Ishii

**Affiliations:** 1 Department of Immunology and Cell Biology, Osaka University Graduate School of Medicine, Suita, Osaka, 565-0871 Japan; 2 Laboratory of Immunology and Cell Biology, WPI-Immunology Frontier Research Center, Osaka University, Suita, Osaka, 565-0871 Japan; 3 Department of General Thoracic Surgery, Osaka University Graduate School of Medicine, Suita, Osaka, 565-0871 Japan; 4 Department of Pathology, Osaka University Graduate School of Medicine, Suita, Osaka, 565-0871 Japan; 5 Laboratory of Bioimaging and Drug Discovery, National Institutes of Biomedical Innovation, Health and Nutrition, Ibaraki, Osaka, 567-0085 Japan; 6 Laboratory of Clinical Pharmaceutical Science, Kobe Pharmaceutical University, Higashinada, Kobe, 658-8558 Japan; 7 Genome Information Research Center, Research Institute for Microbial Diseases, Osaka University, Suita, Osaka, 565-0871 Japan; 8 Laboratory of Human Immunology (Single Cell Genomics), WPI-Immunology Frontier Research Center, Osaka University, Suita, Osaka, 565-0871 Japan

**Keywords:** Lung cancer, Cancer microenvironment, Alveolar macrophages, RNA sequencing

## Abstract

Alveolar macrophages (AMs) are crucial for maintaining normal lung function. They are abundant in lung cancer tissues, but their pathophysiological significance remains unknown. Here we show, using an orthotopic murine lung cancer model and human carcinoma samples, that AMs support cancer cell proliferation and thus contribute to unfavourable outcome. Inhibin beta A (INHBA) expression is upregulated in AMs under tumor-bearing conditions, leading to the secretion of activin A, a homodimer of INHBA. Accordingly, follistatin, an antagonist of activin A is able to inhibit lung cancer cell proliferation. Single-cell RNA sequence analysis identifies a characteristic subset of AMs specifically induced in the tumor environment that are abundant in INHBA, and distinct from INHBA-expressing AMs in normal lungs. Moreover, postnatal deletion of INHBA/activin A could limit tumor growth in experimental models. Collectively, our findings demonstrate the critical pathological role of activin A-producing AMs in tumorigenesis, and provides means to clearly distinguish them from their healthy counterparts.

## Abstract (web summary)

Alveolar macrophages represent a cell type that is physiologic to the lung immune landscape, however, it is not known whether they play an active role to maintain the tumour immune microenvironment. Here authors show by single cell RNA sequencing and functional experiments, that intra-tumour alveolar macrophages are phenotypically and transcriptionally different from the healthy ones, and likely play an aetio-pathologic role in tumorigenesis.

## Introduction

Cancerous tissues comprise a wide variety of cells in addition to tumor cells, such as immune cells, fibroblasts^1^, endothelial cells^2^, and neural cells^3^, which constitute unique microenvironments specific to the cancer cell type. In particular, different types of immune cells have been demonstrated to play critical roles in suppressing or promoting tumor progression in relation to their environment in vivo. Various chemokines secreted by cancer cells mobilize immune cells, such as cytotoxic CD8^+^ T lymphocytes, NK cells, and dendritic cells that attack tumors, into the cancer microenvironment^4^. In contrast, immunosuppressive cell types are also recruited by tumor-secreting cytokines/chemokines, which serve as ‘*internal enemies*’ to promote cancer proliferation, invasion, and metastasis. For example, myeloid-derived suppressor cells (MDSCs) and tumor-associated macrophages (TAMs) are mobilized to the cancer microenvironment via systemic circulation to promote tumor progression^5^. Among them, TAMs are the most abundant, and the majority differentiate from bone marrow-derived Ly6c^+^ inflammatory monocytes^6–8^. TAMs are thought to be influenced by cancer cells after mobilization to transform into phenotypes that benefit the tumor^9^. For instance, TAMs secrete angiogenic factors such as vascular endothelial growth factor to promote tumor angiogenesis and invasion. They also produce transforming growth factor-β (TGF-β) and epidermal growth factor to induce epithelial-mesenchymal transition (EMT) during tumor metastasis. Additionally, TAMs exhibit immunomodulatory properties: TAMs can produce IL-10, TGF-β, and prostaglandin E2; mobilize regulatory T cells (Tregs) via C-C motif chemokine 2 (CCL2); and express programmed death ligand 1/2 (PD-L1/L2) and CD80/86 (B7-1/2) on their cell surfaces to inhibit immune effector cell activation^10^. A comprehensive understanding of tumor-associated immune cells is a prerequisite for the ultimate control of cancer.

Recent studies have revealed that macrophages arise from two distinct lineages, along with the discovery of tissue-resident macrophages (TRMs), which have a different origin from those derived from bone marrow monocytes. In certain tissues, such as the brain, liver, and lungs, TRMs originating from hematopoietic progenitors in the yolk sac at the embryonic stage can maintain themselves in situ by self-renewal and exhibit several microenvironment-specific phenotypes and functions^11–13^. In the lungs, alveolar macrophages (AMs) are TRMs residing in alveolar spaces and constitute one of the two macrophage populations in the lungs, along with interstitial macrophages (IMs) that are mainly of bone marrow origin^14^. AMs have been shown to clean lung surfactants and protect against infection in the homeostatic state^15^. In terms of lung cancers, although the involvement of TAMs has been referred^16^, the possible roles of AMs, even though they are by far abundant major macrophage subsets in cancerous tissues, have seldom been examined. Recently, critical pathological functions of lung TRMs have been suggested based on single-cell RNA sequencing analyses of human non-small cell lung carcinoma (NSCLC) lesions; however, the detailed mechanism of AM-cancer interaction and its clinicopathological relevance remain unclear^17^.

Most of the studies aiming to elucidate cancer-induced host reactions have been done in systems involving ectopically inoculated cancer cells into easily accessible areas, *e.g*. subcutaneous tissues of flanks. In spite of its broad usability, the method does not enable us to examine the actual phenomenon with cancer cells in their unique microenvironments, including possible interactions with residential immune cells.

In this study, by employing an orthotopic lung cancer model, in which cancer cells are surgically implanted into the left lung, we identify residential AMs producing activin A in lung cancer loci as critical players in cancer progression. The data obtained in this more natural experimental model, together with the analytical results arising from studying human samples, suggest an important and targetable role of AMs in lung tumorigenesis.

## Results

### Lung AMs support proliferation of lung cancer cells

**Fig. 1:**
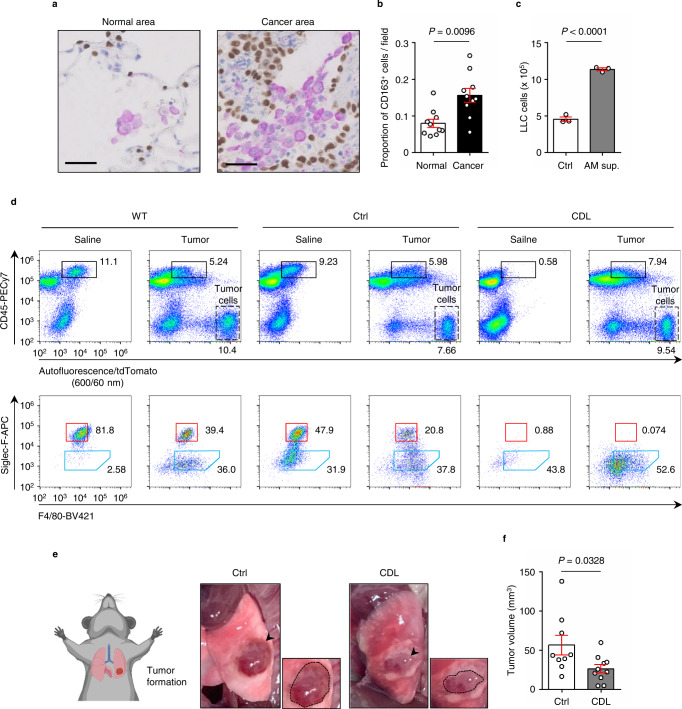
Lung alveolar macrophages (AMs) support proliferation of lung cancer cells. **a** Representative images of immunohistochemical staining of the normal lung area (*left*) and lung cancer area (*right*) from patients with lung cancer. Patient information is listed in Supplementary Table 1. Purple cells indicate CD163^+^ macrophages and brown nuclei indicate alveolar epithelial cells or carcinoma cells positive for thyroid transcription factor-1 (TTF-1). Scale bars; 50 μm. **b** Proportion of CD163^+^ macrophages in total cells within the visual field in normal lung and lung cancer areas, calculated from the mean value of four different visual fields (*n* = 10 patients). **c** Cell count assay. LLC cells were seeded, starved for 24 h, and cultured for 2 days with (or without) AM cell supernatant (*n* = 3 per group). **d** Flow cytometry plots of lung cells from wild-type (WT) C57BL/6 mice, control-liposome (Ctrl) mice, and clodronate liposome (CDL)-treated mice, with or without tumor-bearing conditions. LLC cells was fluorescently labeled with tdTomato (region with a dashed line in the top plots). Bottom plots indicate the analysis of CD45^+^ autofluorescence^+^ cells (black rectangular region in the top plots). The red rectangular region and blue pentagonal region indicate AMs and TAMs, respectively. **e** Tumor formation in the orthotopic lung cancer model. The left image shows a schematic diagram of tumor-bearing mice in supine position. Middle and right images show representative images of the left lung with tumor formation in Ctrl and CDL-treated mice. Arrowheads indicate tumors and small boxes show the enlarged image of the tumor (surrounded by a dashed line). **f** Tumor volume of Ctrl and CDL administration mice (*n* = 9 mice for Ctrl, *n* = 10 mice for CDL). Means ± s.e.m. for each group are shown. Symbols represent individual human patients (**b**), wells in a 6-well plate (**c**), and individual mice (**f**). Statistical significance was determined using paired (**b**) and unpaired (**c**, **f**) two-tailed *t*-tests.

Based on extensive analyses of human clinical histopathological samples of normal and cancerous lung tissues, we observed that CD163-positive AMs accumulated in clusters in cancerous tissues, whereas they were rather sparse in normal alveolar areas (Fig. 1a, Supplementary Table 1). The population of macrophages was significantly increased in cancer tissues compared to those in normal tissues (Fig.1b). These results led us to hypothesize that AMs play a role in the lung cancer microenvironment. Next, we tested the effect of the AM cell line (AMJ2-C11, derived from the C57BL/6 mouse strain). The number of lung carcinoma cells (Lewis lung carcinoma; LLC, derived from the C57BL/6 mouse strain) significantly increased in culture with the AM cell supernatant (Fig.1c); this was associated with a reduced doubling time for the cancer cells (Supplementary Fig. S1a). These results suggested that AMs could influence the proliferation of lung cancer cells via the secreting of soluble factors.

To further analyze the functional roles of lung AMs in cancer proliferation in vivo, we examined an original murine orthotopic lung cancer model. LLC cells stably expressing tdTomato fluorescence were directly inoculated in the left lung (Supplementary Fig. S1b). Lung AMs have been reported to be CD45^hi^, autofluorescence^+^, CD11c^+^, CD11b^−^, Siglec-F^+^, and F4/80^+^^18^. We detected a characteristic CD45^+^ population with strong autofluorescence emission at 600/60 nm wavelength, specifically observed in the lung, but not in the bone marrow or blood (Supplementary Fig. S1c); we defined this population as lung AMs because of their F4/80^+^, Siglec-F^+^, CD11b^−^, and CD11c^+^ characteristics (Fig. 1d and Supplementary Fig. S1d)^19^. Furthermore, lung CD45^+^ autofluorescence^+^ population in LLC-tdTomato-inoculated conditions included not only Siglec-F^+^ F4/80^+^ AMs but also contained a Siglec-F^−^, F4/80^+^, CD11b^+^ population, which could be considered to be TAMs in the lung (Fig. 1d and Supplementary Fig. S1e)^20^. Next, we intratracheally administrated clodronate liposome (CDL), the reagent for macrophage specific depletion^21^, to the mice. CD45^+^ autofluorescence^+^ lung AMs were lost in CDL-treated mice under saline-inoculated conditions, but CD45^+^ autofluorescence^+^ TAMs were retained in tumor-bearing conditions, confirming the specific depletion of lung AMs in CDL-treated mice (Fig. 1d). When inoculating LLCs into the lung tissues of the mice, tumor volumes were significantly smaller in CDL-treated mice than in control-liposome mice (Fig. 1e, f). We found that CDL did not directly affect LLC cell proliferation in vitro compared with the control liposome (Supplementary Fig. S1f). Similar results were obtained from analysis using colony stimulating factor 2 (*Csf2*) knockout mice; Csf2 is critical for differentiation into AMs^22–24^(Supplementary Fig. S1g, h). We confirmed that Csf2 did not directly affect LLC cell proliferation (Supplementary Fig. S1i). We further confirmed that AM depletion via CDL led to metastasis inhibition to contralateral (right side) lung lobes (Supplementary Fig. S1j, k). These results show that AMs in the lung support lung cancer cell proliferation in vivo.

### Inhibin beta A (INHBA)/activin A upregulation in lung AMs enhances proliferation of lung cancer cells in vivo

**Fig. 2:**
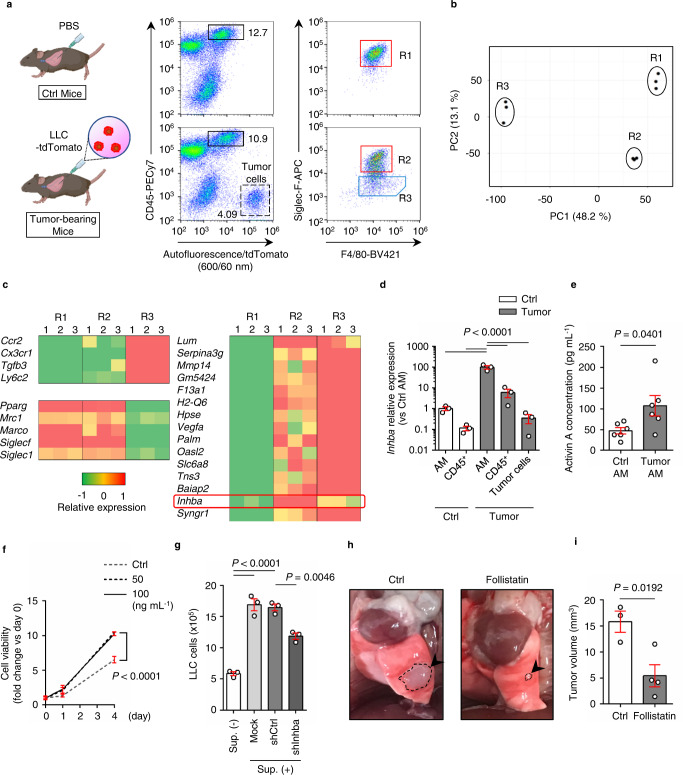
INHBA upregulation in lung alveolar macrophages (AMs) enhances proliferation of lung cancer cells in vivo. **a** Definitions of R1, R2, and R3 cells in the lungs of the control and tumor-bearing mice. Left images show schematic diagram of control (Ctrl) mice with PBS (*upper*) or tumor-bearing mice with LLC cells fluorescently labeled with tdTomato (*lower*). Dot plots on the right indicate the analysis result of CD45^+^ autofluorescence^+^ cells (black rectangular regions in the middle dot plots). The red rectangular region and blue pentagonal region indicate AMs and TAMs, respectively. The data are representative of three independent experiments with similar results. **b** Principal component analysis of R1, R2, and R3 cells by RNA-Seq (*n* = 3 mice for R1, R2, and R3 populations). **c** Heatmaps of tumor-associated macrophage marker genes and AM marker genes (*left*), and the top 15 upregulated genes from R1 to R2 cells (*right*). **d** RT-PCR analysis of *Inhba* expression in AMs and CD45^+^ (autofluorescence^−^) cells isolated from control mice, and AMs, CD45^+^ (autofluorescence^−^) cells, and tumor cells isolated from tumor-bearing mice (*n* = 3 mice per group). **e** Measurement of activin A concentration in AMs sorted from control or tumor-bearing mice using ELISA (*n* = 6 per group). **f** WST-1 cell proliferation assay of LLC cells after administration of each dose of recombinant activin A. Viability of LLC cells in each well with or without recombinant activin A was measured on days 0, 1, and 4. Each value indicates the mean ± s.e.m. of the three wells (*n* = 4 per group for day 0 and day 1, *n* = 3 per group for day 4). **g** Comparison of LLC cell number after 2-day culture with or without conditioned media (Sup.) from shRNA-expressing AM cells (AMJ2-C11) (*n* = 3 per group). **h** Representative images of the left lung of tumor-bearing mice treated with PBS (*left*; control) and follistatin (*right*). Arrowheads indicate the tumor (surrounded by a dashed line). **i** Tumor volume of tumor-bearing mice treated with PBS (control) or follistatin (*n* = 3 mice for control, *n* = 4 mice for follistatin). Means ± s.e.m. for each group are shown. Symbols represent individual mice (**d**, **e**, **i**) or wells (**g**). Statistical significance was determined using one-way ANOVA with Bonferroni’s post hoc test (**d**, **g**) or unpaired two-tailed *t-test* (**e**, **i**).

Next, to examine the molecular mechanism underlying AM-dependent lung cancer progression, we performed comprehensive transcriptome analyses of lung AMs in the presence or absence of cancer cells in vivo using orthotopic lung cancer models. We collected three populations for RNA-sequencing analyses: AMs in control conditions (R1), AMs in LLC-bearing conditions (R2), and TAMs in LLC-bearing conditions (R3) (Fig. 2a). The results of the principal component analysis clearly distinguished the three groups (Fig. 2b). In particular, *Pparg*, which is an important transcription factor for AM, as well as *Mrc1*, *Marco*, *Siglecf*, and *Siglec1* were highly expressed in both R1 and R2, confirming that these are lung AMs^17^. In contrast, R3 highly expressed *Ccr2, Cx3cr1, Tgfb3*, and *Ly6c2*, which are markers for identifying TAMs^6,7,9,25^ (Fig. 2c left). By comparing these three fractions, we observed that *Inhba* is specifically upregulated only in R2 (AMs in tumor-bearing conditions), but not in R1 (AMs in control) or R3 (TAMs), suggesting the specific significance of inhibin beta A (INHBA) in the pathological function of AMs in the tumor environment. (Fig. 2c, right panel).

The INHBA subunit is a member of the TGF-β superfamily that forms homo- or heterodimers with other subunit members (INHBB and INHA) to generate activin and inhibin protein complexes. Quantitative PCR (qPCR) analysis indicated that *Inhba* was significantly and specifically expressed in AMs under tumor-bearing conditions and was absent in control AMs, as well as in other CD45^+^ hematopoietic cell types in the lung (Fig. 2d). Moreover, the orthotopic model established using another lung cancer cell line, KLN205 (derived from the DBA/2 mouse strain), can similarly induce *the Inhba* gene in AMs (Supplementary Fig. S2a). In contrast, the expression levels of other subunit-coding genes, *Inhbb* and *Inha*, did not show any significant changes in AMs under tumor-bearing conditions (Supplementary Fig. S2b). We also confirmed that activin A, the homodimer of the INHBA subunit, is preferentially produced in tumor-bearing AMs (Fig. 2e). To elucidate the functional roles of activin A in lung cancer cells, we treated lung cancer cells with recombinant murine activin A, which resulted in a significant proliferation of LLC cells (Fig. 2f). Furthermore, the cancer proliferation effect using AM cell supernatant in vitro was attenuated by *Inhba* knockdown in AM cells (Fig. 2g, Supplementary Fig. S2c, d). In addition, treatment with follistatin, an antagonist of activin A, significantly reduced the tumor volume in an orthotopic mouse model in vivo (Fig. 2h, i). These results show that activin A secreted by tumor-bearing AMs supports the proliferation of lung cancer cells in vivo.

### Single-cell RNA-seq identified a subtype of tumor-supporting AMs with high expression of INHBA

**Fig. 3:**
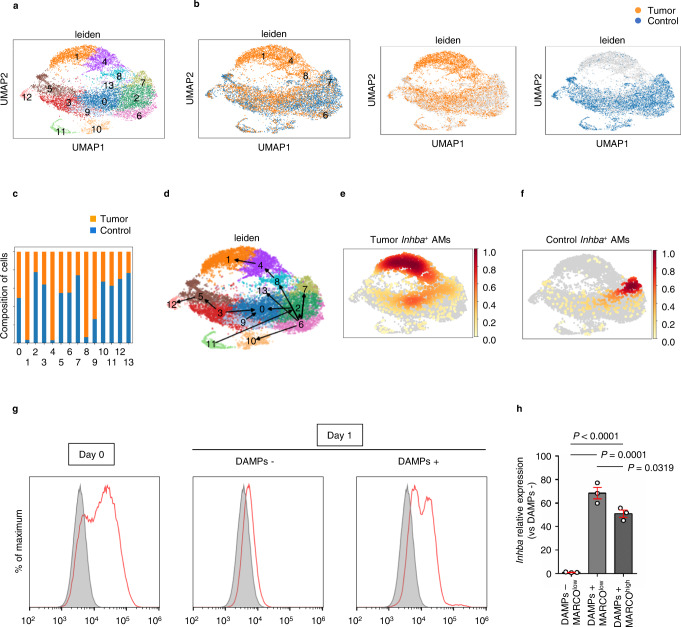
Single-cell RNA-seq identifies a subtype of tumor-supporting alveolar macrophages (AMs) with high expression of INHBA. **a**–**c** UMAP plot of single-cell RNA-seq data of AMs from control and tumor-bearing mice. **a** A total of 13,413 AM cell transcriptomes were analyzed and hierarchically clustered into 14 subgroups using uniform manifold approximation and projection (UMAP) analysis. **b**, **c** Ratio of AMs isolated from control mice (blue) to those isolated from tumor-bearing mice (orange). **d** Schematic image of RNA-velocity analysis results with scVelo. **e**, **f** Distribution of *Inhba* gene expression levels in AMs isolated from tumor-bearing mice (**e**) and those isolated from control mice (**f**), shown using binarization by fitting the expression distribution with Gaussian mixture distribution. **g**, **h** In vitro culture of mouse primary AMs from bronchoalveolar lavage fluid with or without damage-associated molecular pattern molecules (DAMPs) from LLC cells. **g** Flow cytometry histogram of AMs with MARCO. Shaded regions indicate staining with only the second antibody (i.e., without the anti-MARCO antibody). **h** RT-PCR analysis of *Inhba* expression in AMs after in vitro culture for 1 day with or without DAMPs (*n* = 3 per group). Means ± s.e.m. for each group are shown. Symbols represent individual wells (**h**). Statistical significance was determined using one-way ANOVA with Bonferroni’s post hoc test (**h**).

To further characterize tumor-supporting AM populations, we sorted AMs in the orthotopic tumor-bearing model and its control condition and conducted single-cell RNA-sequencing (scRNA-seq) analysis for these populations. A total of 13,413 AM cell transcriptomes were analyzed and hierarchically clustered into 14 subgroups using uniform manifold approximation and projection (UMAP) analysis (Fig. 3a, Supplementary Fig. S3a). We focused on three subclusters: clusters 1, 4, and 8 (Fig. 3b) because all of these were preferentially present in tumor-bearing AMs but not in control AMs (Fig. 3b, c). Cluster 1 was characterized by the expression of *Marco*^low^, *Ly6e*^int^, *S100a6*^high^, and *Cd63*^low^, whereas cluster 4 was characterized by the expression of *Marco*^low^, *Ly6e*^high^, *S100a6*^high^, and *Cd63*^high^, and cluster 8 was characterized by the expression of *Marco*^int^, *Ly6e*^high^, *S100a6*^low^, and *Cd63*^high^, respectively (Supplementary Fig. S3b–i). We further performed RNA-velocity analysis^26^ to estimate the differentiation dynamics of the respective sub-clusters (Fig. 3d). The analysis indicated that cluster 8 was derived from cluster 6 (*Marco*^high^, *Cd9*^high^, and *Ear1*^high^), which was composed of AMs from both control and tumor-bearing conditions (Fig. 3b–d). Furthermore, the analysis indicated that cluster 8 could be differentiated into cluster 1 via cluster 4 (Fig. 3d). Moreover, we checked the expression of *Inhba* in these clusters and found that among AMs from tumor-bearing mice, *Inhba*-high AMs were present in clusters 1, 4, and 8 under tumor-bearing conditions (Fig. 3e), whereas they were present only in cluster 7 under the control condition (*Marco*^int^, *S100a1*^hi^, *Ly6e*^low^) (Fig. 3f) to be derived from cluster 6, as indicated by RNA-velocity analysis (Fig. 3d). Among the genes with significantly higher expression in clusters 1, 4, and 8 (Supplementary Table 2), *Junb* was extracted as the transcription factor with the highest z score (Supplementary Fig. S3j, k). We further examined the effect of tumor-derived factors on AM phenotype in vitro. As measured by flow cytometry, most primary AMs showed high protein expression levels of MARCO in vivo (Fig. 3g, left). However, after 1-day monoculture in vitro these cells became MARCO^low^ (Fig. 3g, middle). After one-day coculture with damage-associated molecular pattern molecules (DAMPs) derived from LLC cells, MARCO^high^ and MARCO^low^ cells were both present (Fig. 3g, right). The coculture with DAMPs induced high expression of *Inhba*, and especially the MARCO^low^ cells showed significant upregulation compared with the MARCO^high^ cells (Fig. 3h). These results showed that under tumor-bearing conditions, some AMs constitute *Inhba*-expressing subclusters through specific differentiation dynamics, which is apparently distinct from *Inhba*-expressing clusters under control conditions.

### INHBA/activin A expression in AMs is induced via MyD88-JNK dependent pathway

**Fig. 4:**
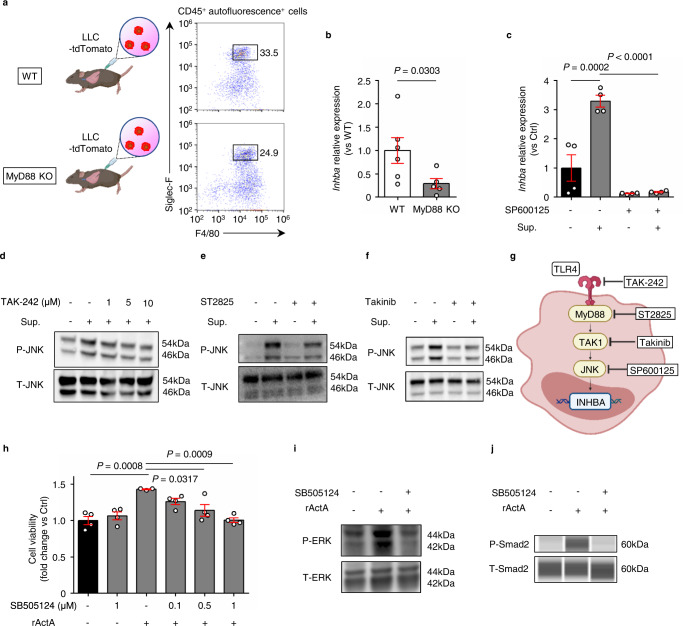
INHBA expression in alveolar macrophages (AMs) is induced via MyD88-JNK dependent pathway. **a** Flow cytometry plots of AMs isolated from C57BL/6 wild-type (WT) mice and MyD88 knockout (KO) mice after inoculation with lung cancer cells. **b** *Inhba* RT-PCR analysis of AM isolated from tumor-bearing wild-type mice and MyD88 knockout mice (*n* = 6 mice for wild-type, *n* = 5 mice for MyD88 KO). **c** *Inhba* RT-PCR analysis in the AM cell line AMJ2-C11 after incubation with or without LLC cell line supernatant (Sup.) and JNK inhibitor SP600125 (*n* = 4 per group). **d**–**f** Immunoblotting of JNK phosphorylation in AM cell line with or without stimulation of LLC cell line supernatant after incubation with each inhibitor (**d**; TAK-242, TLR4 inhibitor, **e**; ST2825, MyD88 inhibitor, **f**; takinib, TAK1 inhibitor). These images were representative of three independent experiments with similar results. **g**, Schematic representation of *Inhba* signaling in AM. **h** Viability of LLC cells treated with the ALK4 inhibitor SB505124 and recombinant activin A (rActA) was assessed using WST-1 assay (*n* = 4 per group except for the one with only rActA administration (*n* = 3)). **i**, **j** Immunoblotting of ERK (**i**) and Smad2 phosphorylation (**j**) in LLC cells stimulated with rActA after incubation with the ALK4 inhibitor SB505124. These images were representative of three independent experiments with similar results. Means ± s.e.m. for each group are shown. Symbols represent individual mice (**b**) and wells (**c**, **h**). Statistical significance was determined using unpaired two-tailed Mann–Whitney *U-*test (**b**) or one-way ANOVA with Bonferroni’s post hoc test (**c**, **h**).

Next, we investigated the molecular mechanism underlying *Inhba* expression in AMs under tumor-bearing conditions. A previous report has indicated that lipopolysaccharide-treated mice show increased activin A through Toll-like receptor 4 (TLR4)-MyD88 signaling^27^. Moreover, it has also been reported that c-Jun N-terminal kinase (JNK) activation is associated with activin A secretion in some cell types^28,29^. Therefore, we established an orthotopic lung cancer model using MyD88 knockout mice and confirmed that the gene expression level of *Inhba* in AMs was significantly decreased in the knockout mice (Fig. 4a, b). Moreover, we found that LLC supernatant-induced upregulated expression of *Inhba* in the AM cell line was canceled by the JNK inhibitor SP600125, suggesting that *Inhba* expression was induced via JNK signaling (Fig. 4c). It has been previously reported that TLR4 is a receptor located upstream of MyD88^30^, and TGF-β-activated kinase 1 (TAK1) functions as an upstream signaling mediator for JNK^31^. Consistent with these reports, we further confirmed that JNK activation in AMs following LLC supernatant administration was inhibited by inhibitors of TLR4, MyD88, and TAK1 (located downstream of MyD88) (Fig. 4d, e, f, Supplementary Fig. S4a–c). These results showed that the TLR4-MyD88-JNK pathway led to *Inhba* expression in AMs (Fig. 4g).

**Fig. 5:**
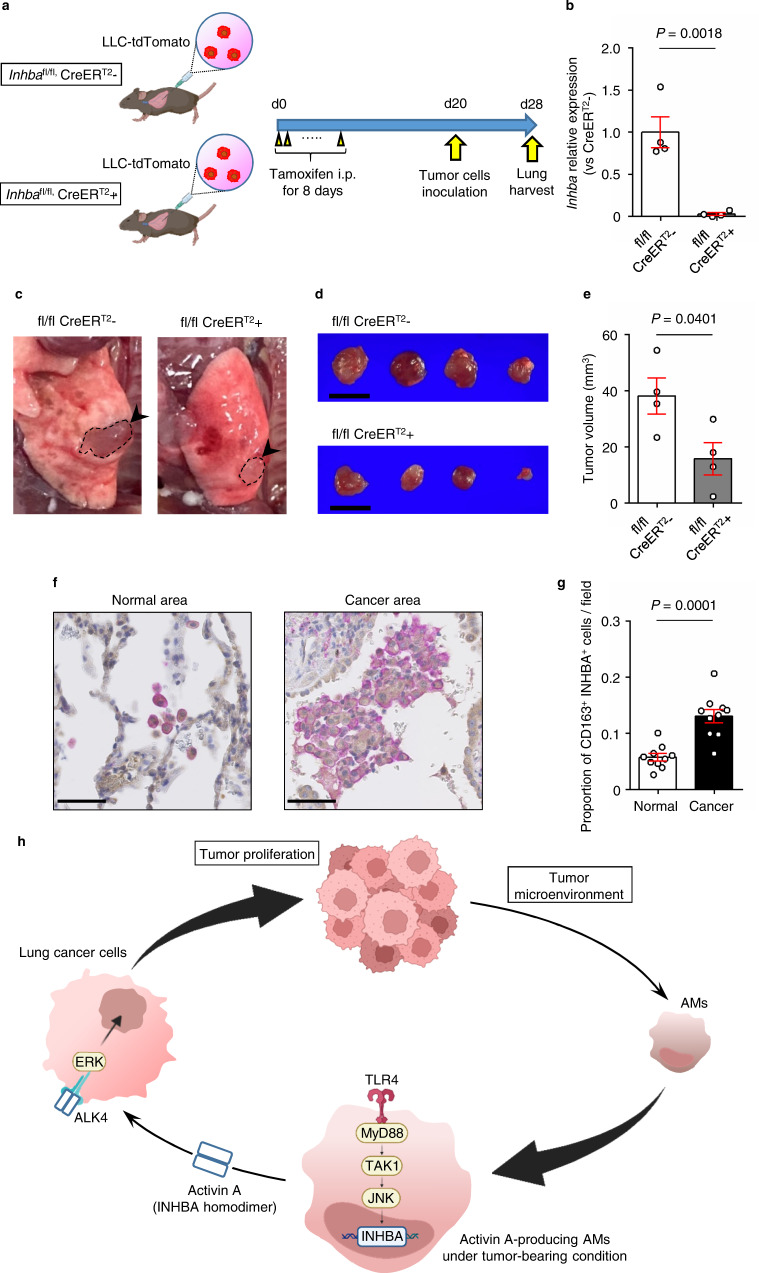
Tumor proliferation is suppressed in INHBA conditional knock out mice. **a** Schematic of the experimental design for tamoxifen-inducible *Inhba* knockout mice with an orthotopic lung cancer model. **b** *Inhba* RT-PCR analysis of AMs isolated from *Inhba*^fl/fl^ CreER^T2^ − and *Inhba*^fl/fl^ CreER^T2^ + mice treated with tamoxifen (*n* = 4 mice per group). **c** Representative images of left lung tumors in tumor-bearing mice. **d** Gross appearance of tumors from *Inhba*^fl/fl^ CreER^T2^ − (*upper*) and *Inhba*^fl/fl^ CreER^T2^ + (*lower*) mice. Scale bars; 5 mm. **e** Tumor volume of *Inhba*^fl/fl^ CreER^T2^ − and *Inhba*^fl/fl^ CreER^T2^ + mice (*n* = 4 per group). **f** Representative images of CD163 (purple) and INHBA (brown) immunostaining of the normal lung area (left) and lung cancer area (right) from patients with lung cancer. Scale bars; 50 μm. **g** Proportion of CD163 + and INHBA + macrophages in total cells within the visual field in normal lung and lung cancer tissues, calculated from the mean value of four different visual fields (*n* = 10 patients). **h** Schematic diagram of the activin A-producing AM subtype in the lung tumor microenvironment. Means ± s.e.m. for each group are shown. Symbols represent individual mice (**b**, **e**) and patients (**g**). Statistical significance was determined using unpaired (**b**, **e**) and paired (**g**) two-tailed *t*-tests.

We further analyzed the growth pathway of LLC cells. The LLC proliferation effect of recombinant activin A was significantly suppressed by SB505124, a competitive inhibitor of activin receptor type-1B (ALK4)^32^ in a dose-dependent manner in vitro (Fig. 4h). In addition, recombinant activin A administration induced phosphorylation of extracellular signal-regulated kinase (ERK) as well as Smad2, which was abolished by SB505124 (Fig. 5i, j). These data indicate that activin A is involved in the proliferation of LLC cells along with ALK4.

### Tumor proliferation was suppressed in INHBA-deficient condition

Next, we analyzed whether specific knock out of the *Inhba* gene induced tumor growth suppression. Since *Inhba* global knockout mice exhibit embryonic lethality^33^, we used *Inhba*-flox mice^34^ and examined the orthotopic lung cancer model using *Inhba*^fl/fl^ *Rosa26*-CreER^T2^ (postnatal *Inhba* deletion) mice (Fig. 5a). Intraperitoneal injection of tamoxifen in *Inhba*^fl/fl^, CreER^T2^ + mice efficiently deleted *the Inhba* gene in AM cells, and qPCR analysis showed that *Inhba* gene expression levels in CreER^T2^ + mice were suppressed 30-fold compared with that in CreER^T2^ − mice (Fig. 5b). Under these conditions, the tumor volume of CreER^T2^ + mice was significantly smaller than that of CreER^T2^ − mice (Fig. 5c–e). Moreover, adoptive transfer of AMs sorted from tumor-bearing CreER^T2^ − mice produced significant tumor enlargement in *Inhba*^fl/fl^, CreER^T2^ + mice (Supplementary Fig. S5a–c). These results indicate that *Inhba*-specific knockout induced tumor growth suppression in vivo.

Finally, we examined whether *Inhba*-high AMs were present in human lung cancer tissues using immunohistochemical staining. More CD163^+^ INHBA^+^ macrophages were observed in the alveolar cavities of cancer tissues than in normal lung tissues (Fig. 5f, g). We further analyzed single-cell transcriptomes from human lung samples using the public dataset deposited in the National Center for Biotechnology Information (NCBI) Gene Expression Omnibus (GEO)^35^. CD45^+^ hematopoietic cells in lung cancer samples and non-involved lung samples were analyzed using Seurat and hierarchically clustered into 20 clusters (cell types) depicted in the UMAP (Supplementary Fig. S5d). We focused on two clusters of *Cd68*^+^ macrophages, clusters 1 and 2, because cells in these clusters expressed *Cd163*, *Siglec1*, and *Marco*, well-defined markers of AMs (Supplementary Fig. S5e). *Inhba*-expressing cells were specifically concentrated in cluster 1 (Supplementary Fig. S5f). Comparing *Inhba* expression in cluster 1 between normal and cancerous samples, we observed significantly higher expression in cancer tissues compared with normal tissues (Supplementary Fig. S5g). This result showed that *Inhba* expression and subsequent activin A production in AMs may influence tumor growth in human lung cancer tissues as well (Fig. 5h).

## Discussion

Despite their abundant presence, the pathophysiological roles of AMs in lung cancer tissues have rarely been reported. A recent study with comprehensive single RNA-sequence analyses of human NSCLC lesions suggested the significance of lung TRMs (including AMs) in tumor growth, especially in the early-stage^17^, although their detailed mechanisms and molecular basis have been left unanswered. In this study, by exploiting original murine orthotropic cancer models as well as human lung cancer specimens, we first showed the critical pathogenic function of lung AMs expressing activin A/INHBA as an inducer of cancer progression. The expression of INHBA in lung AMs is upregulated under tumor-bearing conditions, which in turn supports cancer proliferation, constituting a ‘vicious cycle’ in the tumor environment in vivo.

Activin A is a member of the TGF-β superfamily and was previously identified as a critical factor for ‘activating’ the release of follicle-stimulating hormone (FSH) from the pituitary gland^36^. Thereafter, activin A has been demonstrated to be involved in cellular differentiation and proliferation in a wide array of cell types, both in the developmental and adult stages^37^. There have been several publications on the association between activin A and diverse cancers, and in general, it has been demonstrated that activin A facilitates cancer progression^38^. For instance, activin A has been shown to promote cell invasion and distant metastasis in patients with lung and breast cancer via EMT^39,40^. The pathophysiological significance of upregulation of activin A in tumor-bearing AMs, which was demonstrated in this study, remains unknown, although we suspect that lung AMs may be able to produce activin A in the presence of DAMPs for the purpose of inducing tissue repairs and regenerations^41^. Lung carcinoma cells may misuse the homeostatic system for their survival.

To date, there have been several reports on the presence of ‘disease-specific macrophages’, such as those observed in lung fibrosis^42^ and arthritis^43^. TAMs can also be regarded as members of this population^10^. Characteristically, all of these disease-specific macrophages originate from bone marrow-derived monocytes, migrate into their respective disordered foci, and then become pathogenic cell types in situ^10,42,43^.

Lung cancer is a malignant neoplasm that causes the largest number of deaths worldwide, and it is also one of the most difficult diseases to cure with a 5-year survival rate of ~20%^44^. Recent therapeutic advances, such as those with immune checkpoint inhibitors have been prominent^45^, although the entire treatment strategy still needs to be improved. The incomplete elimination of cancer cells is often attributable to the presence of pro-tumor cell types within the body^46^. The emergence of activin A-producing AMs in lung cancer tissues is one such example. Since follistatin, an activin A inhibitor, significantly blocked tumor progression in vivo, we can reasonably assume that new lines of therapies targeting lung AMs in tumors will become a promising approach for eradicating lung cancers in the future. On the other hand, it remains unknown whether only the tumor-specific *Inhba*-high subset indicated in our RNA-velocity analysis contributes to tumor growth. We think detailed analysis of this subset, such as investigation of master key regulators and cell surface markers, is indispensable to characterize the function of AMs in lung cancer. Moreover, further analysis is also required to elucidate the involvement of AMs in processes other than cancer cell proliferation, such as metastasis, migration, and tumor invasion.

## Methods

### Mice

Wild type C57BL/6 and DBA/2 mice were obtained from CLEA Japan (Tokyo, Japan). Csf2 knock out (B6.129S-Csf2tm1Mlg/J) mice^47^ were obtained from The Jackson Laboratory (Farmington, CT) (Stock Number: 026812). MyD88 knock out (B6.129-Myd88tm1Aki/Obs) mice^48^ were obtained from Oriental Bio Service (Kyoto, Japan) (Stock Number: IMSR_OBS:1). The INHBA LoxP/LoxP^fl/fl^ mice, provided by Prof. N. Emoto (Kobe pharmaceutical University, Kobe, Japan)^34^, were bred with Rosa26CreER^T2^ mice. The mice were bred and maintained under specific pathogen-free conditions at the animal facilities of Osaka University (Osaka, Japan). These mice were maintained in 12 h light/dark cycle, and the housing temperature and humidity were 21.5–24.5 °C and 45–65%, respectively. Mice were given standard laboratory chow diet and water *ad libitum*. All mice used in this study were 8–14 weeks old female. Animal experiments were performed in accordance with the experimental animal guidelines of Osaka University under approved protocols. Mutant mice were genotyped by PCR. To detect the INHBA flox allele, we used the primers ACCCACCGAAGAAGCAAAGA and GGGTCTGAGAGCCCATTGTC.

### Antibodies and reagents

Antibodies were obtained from the following sources: anti-CD45-PECy7 (1:50, clone 30-F11, #103114), anti-F4/80-BV421 (1:50, clone BM8, #123132), anti-CD11c-APC (1:50, clone N418, #117310), anti-CD11b-BV421 (1:50, clone M1/70, #101235), BV421-rat IgG2a, κ isotype control (1:50, clone RTK2758, #400535), and APC-human IgG1, κ isotype control (1:50, clone QA16A12, #403505) were from BioLegend (San Diego, CA); anti-Siglec-F-APC (1:50, clone REA798, #130-112-333), and anti-CD11b-APC (1:50, clone REA592, #130-113-802) were from Miltenyi Biotec (Bergisch Gladbach, NRW, Germany); anti-p-JNK (1:1000, #9251), anti-JNK (1:1000, #9252), anti-p-Erk1/2 (1:1000, #4370), anti-Erk1/2 (1:1000, #4695), anti-p-Smad2 (1:1000, #3108), anti-Smad2 (1:1000, #5339), anti-β-actin (1:1000, #5125), and anti-rabbit IgG, HRP-linked antibody (1:1000, #7074) were from Cell Signaling Technology (Danvers, MA); anti-Inhibin beta A (1:200, ab56057), and anti-TTF-1 (1:100, clone SP141, ab227652) were from Abcam (Cambridge, UK); anti-CD163 antibody (1:800, clone 10D6) was from Leica biosystems (Wetzlar, Germany); anti-MARCO antibody (1:500, clone 2359 A, MAB29561) was from R&D systems (Minneapolis, MN); AlexaFlour647 AffiniPure donkey anti-rabbit IgG (1:200, #711-605-152) was from Jackson Immuno Research (West Grove, PA); and anti-CD16/32 antibody (1:100, clone 2.4G2, #553141) was from BD Biosciences (San Jose, CA). Reagents were obtained from the following sources: Dulbecco’s Modified Eagle’s Medium (DMEM) (4.5 g/L glucose) with L-Gln, without sodium pyruvate (#08459-64), 1× phosphate-buffered saline (PBS) without Calcium and Magnesium (#14249-24), Hank’s balanced salt solution (HBSS) with calcium and magnesium, without phenol red (#09735-75), and RIPA buffer (#16488-34) were from Nacalai Tesque (Kyoto, Japan); Eagle’s Minimum Essential Medium (EMEM) (#30-2003) was from the American Type Culture Collection (ATCC, Manassas, VA); heat-inactivated fetal bovine serum (FBS; #F7524), penicillin/streptomycin (#P4333), and tamoxifen (#T5648) were from Sigma-Aldrich (St. Louis, MO); HEPES (#15630-080), and dispase (#17105-041) were from Gibco (Dublin, Ireland); c-Jun N-terminal kinase (JNK) inhibitor SP600125 (#S1460), TAK1 inhibitor Takinib (#S8663), and ALK4 inhibitor SB505124 (#S2186) were from Selleck Chemicals (Houston, TX); Toll-like receptor 4 (TLR4) inhibitor TAK-242 (#HT-11109), and MyD88 dimerization inhibitor ST2825 (#HY-50937) were from MedChemExpress (Monmouth Junction, NJ); recombinant human/mouse/rat activin A protein (#338-AC) was from R&D Systems (Minneapolis, MN); recombinant human follistatin (#120-13), and recombinant murine GM-CSF (#315-03) were from Peprotech (Cranbury, NJ); clodronate liposome (MKV100) and equivalent control liposome were from CosmoBio (Tokyo, Japan); Hoechst 33342 (#3570 invitrogen) was from Thermo Fisher Scientific (Waltham, MA); collagenase type IV (#LS004188) was from Worthington Biochemical Corporation (Lakewood, NJ); PhosSTOP (#4906845001), and EDTA-free Protease Inhibitor Cocktail (#4693159001) were from Roche (Basel, Switzerland).

### Cell culture

Lewis lung carcinoma (LLC), KLN205, and AMJ2-C11 (alveolar macrophage cell line) were purchased from ATCC. LLC cell was maintained in DMEM with 10% FBS and penicillin/streptomycin, KLN205 cell was maintained in EMEM with 10% FBS and penicillin/streptomycin, and AMJ2-C11 cell was maintained in DMEM with 5% FBS, 5 µM HEPES, 10 ng/mL of GM-CSF and penicillin/streptomycin, respectively. To generate LLC cells stably expressing tdTomato (LLC-tdTomato), tdTomato (ptdTomato-C1; #632533; Takara Bio USA, Mountain View, CA) was inserted into the CSII-EF-MCS vector (provided by Dr. Miyoshi of RIKEN-BRC, Tsukuba, Japan) and transfected into HEK293T cells (ATCC) with packaging plasmids. Two days after inoculation, LLC cells were incubated with lentiviral supernatant from virus-producing HEK293T cells, together with polybrene (8 µg/mL) for 12 h. To generate AMJ2-C11 cells expressing short hairpin (sh) RNAs, pooled sh-*Inhba* (TR516955, Origene, Rockville, MD) with sequences GAGGAGTGAACTGTTGCTATCAGAGAAAG, CCTTGCTTTGGCTGAGAGGATTTCTGTTG, CTCGCTCTCCTTCCACTCAACAGTCATTA, and GGATTGCTTGTGAGCAGTGCCAGGAGAGT, or scrambled shRNA (TR30012, Origene) were transfected into Plat-E cells with TurboFectin 8.0 Transfection Reagent (TF81001, Origene). Two days after transfection, AMJ2-C11 cells were incubated with retroviral supernatant from virus-producing Plat-E cells, together with polybrene. Stable transformants were selected using an SH800 cell sorter (Sony, Tokyo, Japan).

### Cell proliferation assay

LLC cells were seeded in 96-well plates at 2 × 10^3^ cells/well, starved for 24 h, then used in cell proliferation assays. The indicated concentration of ALK4 inhibitor SB505124 or recombinant activin A was added into the culture medium and incubated for the same time as the control group. The culture medium was changed on days 2 and 4. Cell viability was determined at the indicated time points using a water-soluble tetrazolium salts (WST-1) assay according to manufacturer’s protocol (Roche Applied Science, Mannheim, Germany). Briefly, after incubation with 10 µL of WST-1 solution for 3 h at 37 °C, the absorbance at 450 nm in each well was determined with a fluorescence microplate reader (SH-9000Lab; Hitachi High-Tech Science Corporation, Tokyo, Japan). Cell viability was calculated based on comparison with the absorbance of control group.

Conditioned medium (CM) was obtained from LLC and AM cells. 3 × 10^6^ LLC cells were seeded in 10-cm dish and incubated in DMEM with 10% FBS for 24 h. Attached cells were washed once with phosphate-buffered saline, and incubated with serum-free DMEM for 3 days. The supernatant was collected after centrifugation at 300 × g. LLC-CM was used for quantitative reverse transcription-polymerase chain reaction (RT-PCR) analysis and immunoblotting analysis. 1 × 10^5^ AM cells were seeded in 6 well plate and incubated in DMEM with 5% FBS for 24 h. Attached cells were washed once with 1× PBS, and incubated with serum-free DMEM for one day. The supernatant was collected after centrifugation at 200 x g for 5 min, and filtered through a 0.22 μm syringe filter. AM-CM was applied to LLC cells after seeding 1 × 10^4^ cells in 6 well plate, incubating for 2 days. Then, LLC cells were counted using TC10 Automated Cell Counter (Bio-Rad, Hercules, CA).

### Orthotopic lung cancer model and chemical administration

The orthotopic lung cancer model was generated by implanting murine lung cancer cells into the left lung of mice as previously described^49^. Mice were anesthetized with isoflurane, and placed in the right lateral decubitus position. A small skin incision to the left chest wall was made ~10 mm, parallel to ribs. 1 × 10^6^ tumor cells suspended in 10 μL of 1× PBS and 10 μL of Matrigel (#356234, Corning, Corning, NY) were directly injected through the intercostal space into the left lung to a depth of 2 mm using a 30 G needle attached to a 0.5 mL insulin syringe (BD Biosciences). The skin incision was closed using a 4–0 silk suture. Experimental mice and control ones were bred separately. Tumor volumes of the compared mice were measured after the same period (8–15 days) after inoculation according to the formula of the length × width^2^ × 0.5. The maximal tumor burden permitted by the ethics committee was the diameter of 2 cm, which was not exceeded in this study. In addition to the above, water intake disorders, eating disorders, and weight loss of 20% or more were also set as endpoints, but none of these were applicable in this study. Mice were euthanized by carbon dioxide inhalation before lung extraction. For Activin A antagonist treatment, one day before the cancer cells were implanted, mice were treated with 50 µg/kg follistatin intraperitoneal injection every day from one day before the inoculation. For the AM depletion model, the clodronate liposome (25 mg/kg) or an equivalent control liposome were administrated intratracheally 2 days before the tumor inoculation and two additional times more than 3 days after the inoculation (75 mg/kg in total).

### Flow cytometry analysis and cell sorting

Blood sample was collected from the abdominal vena cava after euthanasia. The lungs from female adult mice were harvested immediately without perfusion and stored in 1× PBS in 1.5 mL safe-lock tubes on ice. The lung tissues were minced with autoclaved scissors for about 2 min, and digested with 1 mg/mL collagenase type IV and 3 mg/mL dispase in HBSS at 37 °C for 45 min. For the coculture analysis with DAMPs, primary AMs were collected from wild type C57BL/6 mouse bronchoalveolar lavage fluid similarly to a previous report^50^. DAMPs were extracted from LLC cells also in accordance with previously reported methods^51^. In brief, LLC cells were suspended at 10^8^ cells/mL and supernatant was collected via centrifugation at 300 × g for 5 min after being subjected to five freeze-thaw cycles. Bone marrow samples were harvested from femur and tibia bones and crushed in fluorescence-activated cell sorting (FACS) buffer (1× PBS, 4% FBS, and 2 mM EDTA). Disaggregated tissue elements were passed through a 70-µm cell strainer and centrifuged at 300 × g for 5 min to prepare single cell suspensions. Zombie Green (#423111, BioLegend) was used to remove dead cells. Blood and isolated cells were blocked with anti-CD16/32 antibody for 15 min, followed by staining with the antibodies described above for 30 min. Measurements and cell sorting were performed on an SH800 cell sorter (Sony) and analyzed with FlowJo v.10 (FlowJo; LLC). When analyzing tumor cell metastasis to contralateral lung lobes, positivity for metastasis was defined as the lobe containing more than 0.01% of tumor cells (CD45^−^, tdTomato^+^ cells).

### RNA sequencing

AMs from orthotopic vehicle-control or lung cancer model mice were isolated using an SH800 cell sorter, and total RNA was extracted using QIAzol lysis reagent (#79306; Qiagen, Germantown, MD), in accordance with the manufacturer’s instructions. Total RNA was extracted from cells with an miRNeasy Mini kit (#217004; Qiagen) according to the manufacturer’s protocol. Full-length cDNA was generated using a SMART-Seq HT Kit (#634455; Takara Bio, Mountain View, CA) according to the manufacturer’s instructions. An Illumina library was prepared using a Nextera XT DNA Library Prep Kit (Illumina, San Diego, CA) according to SMARTer kit instructions. Sequencing was performed on an Illumina HiSeq2500 sequencer (Illumina) in the 75-base single-end mode. Sequenced reads were mapped to the mouse reference genome sequences (mm10) using TopHat v2.1.1 in combination with Bowtie2 ver. 2.2.3 and SAMtools ver. 1.8. The fragments per kilobase of exon per million mapped fragments (FPKMs) was calculated using Cufflinks version 2.2.1. Bioinformatics analyses were performed using Integrated Differential Expression and Pathway Analysis (iDEP) v. 0.81, and Ingenuity Pathway Analysis software (Ingenuity Systems; Qiagen). The raw reads from the sample have been deposited in the NCBI Gene Expression Omnibus database (GEO GSE193913).

### Preparation for single-cell RNA-Seq analysis

FACS-isolated AM cells from orthotopic vehicle-control or lung cancer model mice were used for single-cell RNA-Seq. Single-cell RNA library construction and sequencing Single-cell suspension were processed through the 10x Genomics Chromium Controller following the protocol outlined in the Chromium Single Cell 3' Reagent Kits User Guide. Chromium Next GEM Single Cell 3' Kit v3.1 (#PN-1000269; 10x Genomics, Plesanton, CA), Chromium Next GEM Chip G Single Cell Kit (#PN-1000127; 10x Genomics) and Dual Index Kit TT Set A (#PN-1000215; 10x Genomics) were applied during the process. Approximately 10,000 live cells per sample, according to the manufacturer’s recommendations, were loaded onto the Chromium controller to generate 7000 single-cell gel-bead emulsions for library preparation and sequencing. Oil droplets of encapsulated single cells and barcoded beads (GEMs) were subsequently reverse-transcribed in a Veriti Thermal Cycler (Thermo Fisher Scientific), resulting in cDNA tagged with a cell barcode and unique molecular index (UMI). Next, cDNA was then amplified to generate single-cell libraries according to the manufacturer’s protocol. Quantification was made with an Agilent Bioanalyzer High Sensitivity DNA assay (#5067-4626; Agilent, Santa Clara, CA). Subsequently amplified cDNA was enzymatically fragmented, end-repaired, and polyA tagged. Cleanup/size selection was performed on amplified cDNA using SPRIselect magnetic beads (#B23317, Beckman-Coulter, Brea CA). Next, Illumina sequencing adapters were ligated to the size-selected fragments and cleaned up using SPRIselect magnetic beads. Finally, sample indices were selected and amplified, followed by a double-sided size selection using SPRIselect magnetic beads. Final library quality was assessed using an Agilent Bioanalyzer High Sensitivity DNA assay. Samples were then sequenced on an Illumina NovaSeq 6000 as paired-end mode (read1: 28 bp; read2: 91 bp). The resulting raw reads were processed by cellranger 5.0.0 (10x Genomics). The raw reads from the sample have been deposited in the NCBI Gene Expression Omnibus database (GEO GSE 193914).

### RNA-velocity analysis

The single cell RNA-Seq fastq files were processed through 10x Genomics Cell Ranger 5.0.0^52^ with default settings, resulting in gene expression profiles of hashtags attached cell barcodes. The resulting alignment files were then processed with velocyto^26^ for the determination of nascent (unspliced) and mature (spliced) mRNA spanning reads abundance. Transcriptomes of the 8485 and 9175 barcodes from two different libraries were merged and conducted with downstream analysis with Scanpy^53^. Read counts of the hashtags of the cell barcodes were extracted and scaled from 0 to 10. Cell barcode originated from which sample was then estimated based on the hashtag read counts of each barcode. To avoid potential bias of the clustering results, ribosome genes, TRAV, TRAJ, TRBJ, TRBV, IGHV, IGKV and IGLV genes were excluded in the downstream analysis. Scrublet^54^ was applied to predict and filter potential doublets. Distribution of the read counts, count of gene per barcode, ribosome gene concentration, mitochondria gene concentration and hemoglobin gene concentration within the sample were each fitted with mixture of two Gaussian distributions^55^. The filtration thresholds for the quality control of the cell barcodes were then determined by the distance of the fitted mean values of the Gaussian distributions. Resulting 13,413 cells consist of 7368 cells from Tumor-AM sample, and 6045 cells from the Control-AM sample were used in the following analysis. Batch effect correction were conducted through Scanorama^56^. Leiden clustering^57^ and PAGA graph^58^ were integrated with UMAP projection^59^. Among the resulting 16 clusters (from 0 to 15), cells from the smallest two cluster 14 and 15 were considered as not related cells and excluded. 13,358 cells used in the final analysis. RNA-velocity analysis was conducted with scVelo^60^.

### Quantitative reverse transcription-polymerase chain reaction (RT-PCR)

Total RNA and cDNA were prepared using RNeasy Mini kit and Micro kit (#74104 and #74004, Qiagen, Venlo, Netherland), and Superscript III reverse transcriptase (Thermo Fisher Scientific), according to the manufacturers’ instructions. Quantitative RT-PCR was performed with a Thermal Cycler Dice Real-Time System (TaKaRa Bio, Shiga, Japan) using SYBR Premix EX Taq (TaKaRa Bio). The reactions were normalized relative to the housekeeping gene *Gapdh*. The following specific primer pairs were used (forward and reverse, respectively): *Gapdh* (5′-TGTGTCCGTCGTGGATCTGA-3′ and 5′-CCTGCTTCACCACCTTCTTGAT-3′); *Inhba* (5′-GGAGAACGGGTATGTGGAGA-3′ and 5′-TGGTCCTGGTTCTGTTAGCC-3′); *Inhbb* (5′-AGGCAACAGTTCTTCATCGACTTTC-3′ and 5′-AGCCACACTCCTCCACAATCATG-3′); *Inha* (5′-TTCATTTTCCACTACTGCCATGGTA-3′ and 5′-GATACAAGCACAGTGTTGTGTAATG-3′). To detect the deletion of *Inhba*, we used the following primers: (5′-GAAGGCAACCACACGACTTTTGCTGC-3′ and 5′-CTCTGGCTGAGAGTTAGGTCCATCCTTC-3′).

### Enzyme‑linked immunosorbent assay (ELISA)

AMs from orthotopic vehicle-control or lung cancer model mice were isolated by FACS and incubated in 96-well plate overnight. Activin A protein levels in cell culture supernatants were determined using the Activin A ELISA kit (#OKBB00124, Aviva Systems Biology, San Diego, CA) according to the manufacturer’s instructions.

### Immunoblotting

Cells were rinsed with ice-cold PBS and lysed in RIPA buffer containing PhosSTOP and EDTA-free Protease Inhibitor Cocktail for 10 min. The soluble fractions from the cell lysates were isolated by centrifugation at 4 °C for 10 min at 13,400 × g. Next, 2×SDS buffer (4% SDS, 125 mM Tris-HCl pH 6.8, 20% glycerol, 0.01% bromophenol blue, 10% 2-mercaptoethanol) was added to the cell lysates. Protein concentrations were measured using Quick Start^TM^ Bradford Protein Assay Kit 2(#5000202JA, Bio-Rad). Proteins were analyzed by SDS–polyacrylamide gel electrophoresis and immunoblotting following standard protocols using anti-p-JNK, anti-JNK, anti-p-Erk1/2, and anti-Erk1/2 antibodies (1:1000 dilution each). Finally, bands were visualized with the enhanced chemiluminescence reagents (Perkin Elmer, Waltham, MA) and using an ImageQuant LAS-4000 mini system (GE Healthcare, Chicago, IL). Quantification was performed with Image J software. Protein detection of p-Smad2 and Smad2 was performed with WES 004-600 (Bio-Techne, Minneapolis, MN) according to the manufacturer’s instructions.

### Evaluation of postnatal *Inhba* deletion mice

To induce *Inhba* conditional knockout mice, the INHBA^fl/fl^ mice were bred with Rosa26CreER^T2^ mice. INHBA^fl/fl^/Cre^−^ or INHBA^fl/fl^/Cre^+^ mice were treated with 200 µL tamoxifen i.p. daily for eight consecutive days. On 20 days after the first dose of tamoxifen, 1 × 10^6^ LLC cells per mouse were inoculated into the left lung, followed by measurement of gene expression level of *Inhba* and tumor volume on day 28. For the adoptive transfer of AMs, 1 × 10^5^ AM cells (CD45^+^, autofluorescence^+^, Siglec-F^+^, and F4/80^+^) were collected via FACS from the lungs of tumor-bearing INHBA^fl/fl^/Cre^−^ mice and co-inoculated with LLC cells into an INHBA^fl/fl^/Cre^+^ mouse.

### Surgical specimens from patients with lung cancer

Cancer tissue or normal lung tissue far from cancer lesion were obtained from the patients who underwent surgical resection at Osaka University Hospital. These tissue specimens were fixed in 10% formalin and processed routinely for paraffin embedding. This study was conducted in accordance with the Declaration of Helsinki (revised in 2013) and approved by the Institutional Review Board of Osaka University on June 14, 2019 (Approval No. 18518). Need for individual consent was waived, as this was a retrospective analysis and data were accessed after masking patients’ identity.

### Immunohistochemistry

Paraffin-embedded tissues were sectioned, processed and subjected to immunohistochemistry using Dako Autostainer Link 48 (Agilent, Santa Clara, CA) with an anti-Inhibin beta A antibody (1:200 dilution), anti-CD163 antibody (1:800 dilution), and anti-TTF-1 antibody (1:100 dilution). 3,3'Diaminobenzidine (DAB) (Agilent) and Stayright Purple (#45906, AAT Bioquest, Sunnyvale, CA) were used as the chromogens. Sections were counterstained with hematoxylin for 1 min before mounting. The proportions of CD163^+^ cells or CD163^+^ INHBA^+^ cells in the alveolar cavity were calculated by counting the cells of interest in four visual fields (square with 225 µm side each) within the section, as well as total cell number in the visual fields using HALO v3.5.3577 software (Indica Labs, Albuquerque, NM).

### Confocal microscopy imaging

The imaging for metastatic foci in the contralateral lung lobe was performed using an A1 confocal microscope system (Nikon, Tokyo, Japan). Lung tissues were stained with Hoechst 33342 (1:1000) on ice for 20 min before imaging. Raw imaging data were processed using NIS-Elements software (Nikon).

### Reanalysis of human lung scRNA-seq data

We obtained an scRNA-seq dataset of CD45^+^ cells from ten patients (five normal and five cancerous lungs) from the GEO (“GSE154826”)^35^. We used R (version 4.1.2) and Seurat (version 4.1.1) to process the data^61^. Next, we performed unsupervised clustering and gene expression analysis according to the Seurat guidance. In brief, we first removed genes detected in fewer than five cells in a sample, cells with fewer than 200 or >5000 genes and cells with >20% mitochondrial genes from the data. The resulted in 62,188 cells, 31,168 cells from the normal samples and 31,020 cells from the cancerous samples; these cells were used in the following analysis. All data were integrated using the reciprocal principal components analysis (RPCA)-based integration method. We then performed cluster classification using the nearest neighbor graph-based clustering method, in which we tuned the resolution parameter to determine the number of clusters.

### Statistical analysis

Data represent means ± standard errors of the mean (s.e.m.). A two-tailed *t*-test, Mann–Whitney *U*-test or Fisher’s exact test were used for comparisons between two groups. Statistical significance was calculated using one-way analysis of variance (ANOVA) with Bonferroni’s post hoc test or the Tukey−Kramer post hoc test for comparisons among three or more groups. Statistical analyses were performed using Graphpad Prism v.7 software (GraphPad Software, San Diego, CA) according to the manufacturer’s instructions.

### Reporting summary

Further information on research design is available in the Nature Portfolio Reporting Summary linked to this article.

## Supplementary information

Supplementary Information

Reporting Summary

## Data Availability

The datasets analyzed during the current study are available from the corresponding authors on reasonable request. Source data are provided with this paper. The raw reads of RNA-sequencing analyses from mice macrophages have been deposited in the NCBI GEO database under accession number and hyperlinks: “GSE193913”. The raw reads of single-cell RNA-seq analysis from mice AMs have been deposited in the NCBI GEO database (“GSE193914”). Source data are provided with this paper.

## Source data

Source Data

## Code availability

All source codes are deposited in the GitHub repositories [https://github.com/liuifrec/Taniguchi_manuscript_notebooks] and [https://github.com/OU-ICB/2022_STaniguchi].
