# Supplementary material for: In vivo induction of activin A-producing alveolar macrophages supports the progression of lung cell carcinoma
Source: Nat Commun. 2023 Jan 17;14:143. doi: 10.1038/s41467-022-35701-8 (PMC9845242; doi:10.1038/s41467-022-35701-8)
Supplement: Supplementary file 1 — Supplementary Information [file 41467_2022_35701_MOESM1_ESM.pdf]

Supplementary Data

Title: In vivo induction of activin A-producing alveolar macrophages supports the progression of lung cell carcinoma

Author List: Seiji Taniguchi[1,2,3], Takahiro Matsui[1,4,*], Kenji Kimura[3], Soichiro Funaki[3], Yu Miyamoto[1,2], Yutaka Uchida[1,2], Takao Sudo[1,2], Junichi Kikuta[1,2,5], Tetsuya Hara[6], Daisuke Motooka[7,8], Yu-Chen Liu[8], Daisuke Okuzaki[7,8], Eiichi Morii[4], Noriaki Emoto[6], Yasushi Shintani[3], and Masaru Ishii[1,2,5,*]

Affiliations:

1: Department of Immunology and Cell Biology, Osaka University Graduate School of Medicine, Suita, Osaka 565-0871, Japan.

2: Laboratory of Immunology and Cell Biology, Immunology Frontier Research Center, Osaka University Graduate School of Medicine, Suita, Osaka 565-0871, Japan

3: Department of General Thoracic Surgery, Osaka University Graduate School of Medicine, Suita, Osaka 565-0871, Japan.

4: Department of Pathology, Osaka University Graduate School of Medicine, Suita, Osaka 565-0871, Japan.

5: Laboratory of Bioimaging and Drug Discovery, National Institutes of Biomedical Innovation, Health and Nutrition, Ibaraki, Osaka 567-0085, Japan

6: Laboratory of Clinical Pharmaceutical Science, Kobe Pharmaceutical University, Higashinada, Kobe 658-8558, Japan

7: Genome Information Research Center, Research Institute for Microbial Diseases, Osaka University; Suita, Osaka 565-0871, Japan.

8: Laboratory of Human Immunology (Single Cell Genomics), WPI Immunology Research Center, Osaka University, Suita, Osaka 565-0871, Japan.

* Corresponding authors

Supplementary Information

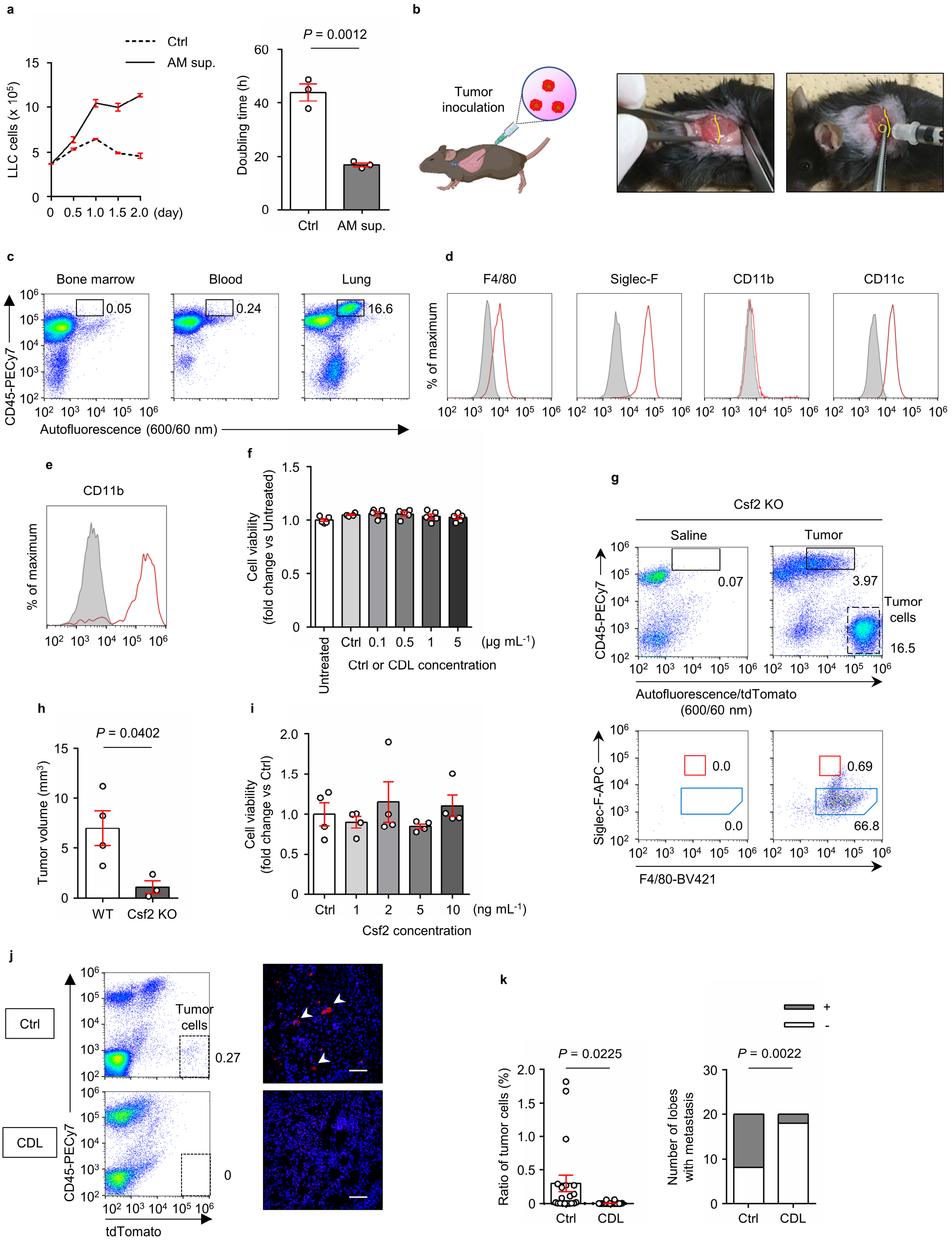

Supplementary Fig. S1. Lung alveolar macrophages (AMs) support proliferation of

lung cancer cells.

a, Cell count assay for doubling time analysis. LLC cells were seeded, starved for 24 h, and cultured in the presence or absence of AM cell supernatant for 2 days (*left*, n = 3 per group). Doubling times were calculated using the counts from 0.5 and 1.0 days, considered the exponential growth phase (*right*, n = 3 per group). **b**, Establishment of an orthotopic lung cancer model. The image on left shows a schematic diagram of a mouse with inoculated tumor cells in a lateral supine position, and images on right show the picture of a mouse model with exposure of thoracic cavity. Yellow line represents the diaphragm and yellow circle indicates the point of tumor inoculation. **c-d**, Flow cytometry plots of bone marrow, blood, and lung cells from C57BL/6 wild type mouse (**c**), and flow cytometry-based phenotypic characterization of $CD45^+$ autofluorescence$^+$ cells in the lung (black rectangle region of the right plot in **c**) with the indicated cell-surface markers (**d**). Shaded regions indicate staining with isotype controls. **e**, Flow cytometry histogram of TAM population ($CD45^+$, autofluorescence$^+$, Siglec-F$^-$ cells) with CD11b. Shaded regions indicate staining with isotype control. **f**, Cell viability of LLC cells with administration of each dose of clodronate liposome (CDL) or control liposome (Ctrl). Cell viability was measured using the WST-1 assay (n = 4 for Ctrl, n = 5 for other groups). **g**, Flow cytometry plots of lung cells from Csf2 knockout (KO) mice, with or without tumors. Plots in bottom panel indicate the analysis of $CD45^+$

autofluorescence^{+} cells (black rectangular region in the top plots). The red rectangular and blue pentagonal regions indicate AMs and TAMs, respectively. **h**, Tumor volume of wild-type (WT) and Csf2 KO mice (n = 4 mice for WT, n = 3 mice for Csf2 KO). **i**, Cell viability of LLC cells with administration of each dose of recombinant csf2. (n = 4 per group). **j**, Flow cytometry plots (*left*) and confocal microscopy images (*right*) of cells in contralateral lung lobes from Ctrl mice and CDL-treated mice, with tumor-bearing condition. In confocal microscopy imaging, live cells were stained with Hoechst 33342 (blue). White arrowheads indicate tdTomato^{+} LLC cells (red). Scale bars, 100 µm. **k**, Quantitative comparison of contralateral lung metastases. The proportion of tumor cells (CD45^{-}, tdTomato^{+} cells) was calculated in every lobe of the contralateral (right side) lung (*left*, n = 20 lobes per group). Means ± s.e.m. for each group are shown. Statistical significance was determined using unpaired two-tailed *t-test* (**a, h, k** left), Fisher's exact test (**k** right) or one-way ANOVA with Bonferroni's post hoc test (**f**, **i**).

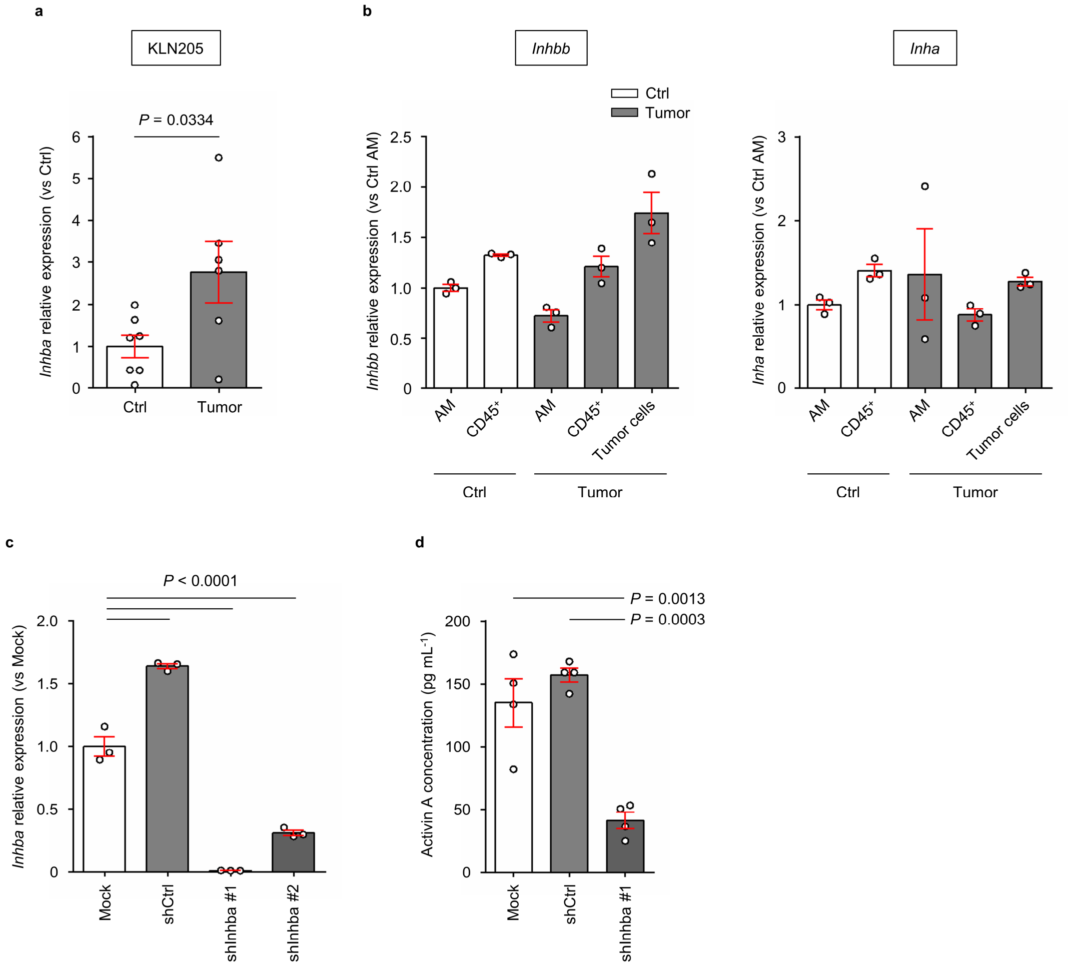

Supplementary Fig. S2. INHBA upregulation in lung alveolar macrophages (AMs) enhances proliferation of lung cancer cells.

a, RT-PCR analysis of *Inhba* expression in AM cells isolated from DBA/2 control mice and those isolated from DBA/2 tumor-bearing mice inoculated with KLN205 cells (n = 7 mice for control, n = 6 mice for tumor-bearing group). **b**, RT-PCR analysis of *Inhbb* and *Inha* expression in AMs and $CD45^+$ cells isolated from control mice, and AMs, $CD45^+$ cells and tumor cells isolated from tumor-bearing mice (n = 3 mice for per group). **c**, RT-PCR analysis of *Inhba* expression in AM cells (AMJ2-C11) with or without shRNA transfection. Two clones

(#1, 2) of AM cells transfected with the sh-*Inhba* gene were analyzed. (n = 3 per group). **d**, Measurement of activin A concentration in AMs transfected with or without shRNA using ELISA (n = 4 per group). Clone #1 in Supplementary Fig.S2c was used for the analysis. Means ± s.e.m. for each group are shown. Statistical significance was determined using unpaired two-tailed *t* test (**a**) and one-way ANOVA with Bonferroni's post hoc test (**b-d**).

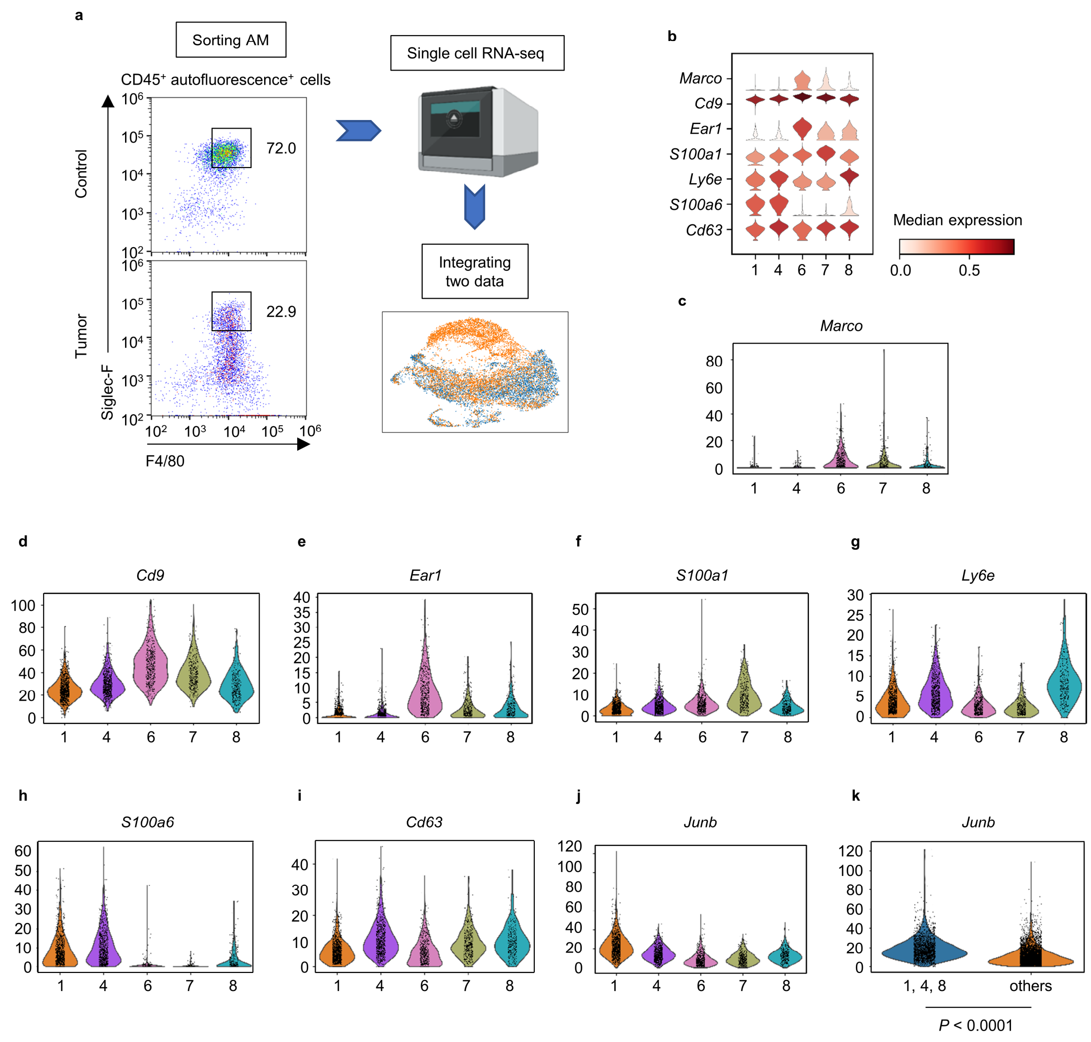

Supplementary Fig. S3. Single-cell RNA-seq identifies a subtype of tumor-supporting alveolar macrophages (AMs).

a, Schematic protocol of the single cell RNA-seq analysis. Alveolar macrophages (AMs) were sorted from control mouse or tumor-bearing mouse. **b**, Median expression levels of the assigned markers: *Marco*, *Cd9*, *Ear1*, *S100a1*, *Ly6e*, *S100a6*, *Cd63*. **c–j**, Violin plots show the expression level of genes in clusters 1, 4, 6, 7, and 8. Each dot represents a single cell. **k**, Violin plot showing the expression level of the *Junb* gene in 3 clusters (1, 4, and 8)

compared with the other 11 clusters. Statistical significance was determined using two-tailed Mann-Whitney *U* test (**k**).

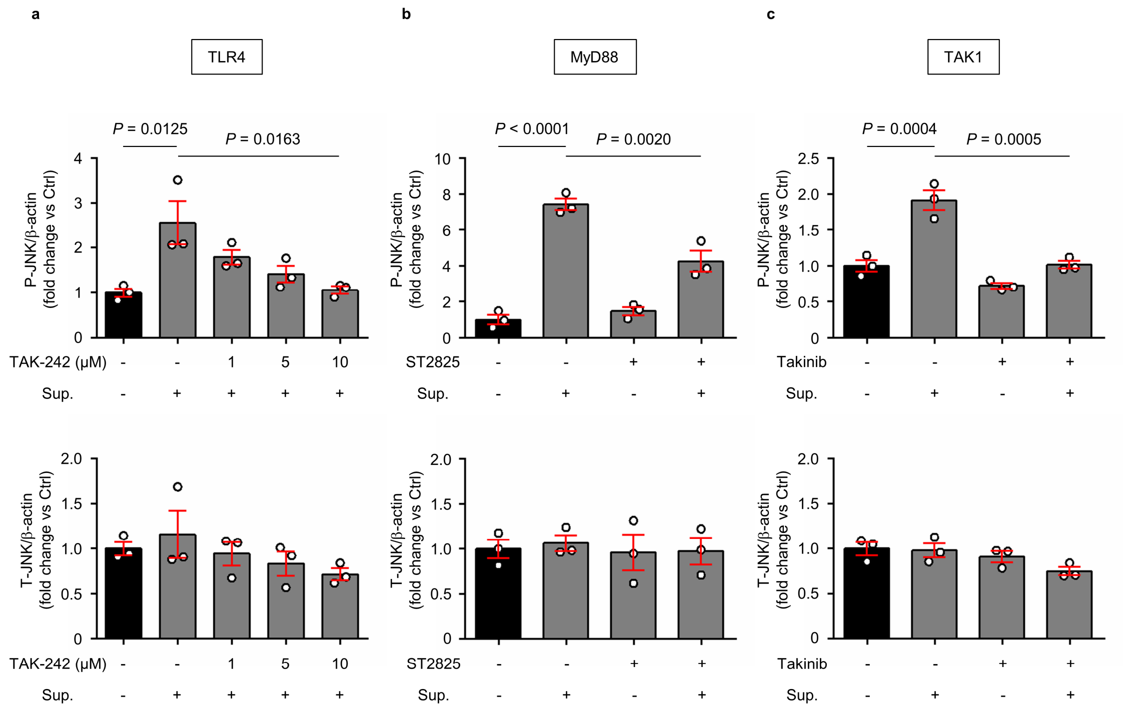

Supplementary Fig. S4. INHBA expression in alveolar macrophages (AMs) is induced via MyD88-JNK dependent pathway.

a-c, Quantification of immunoblotting images using Image J software (n = 3 per group). The samples derive from the same experiment and the gels were processed in parallel. Statistical significance was determined using one-way ANOVA with Bonferroni's post hoc test. Means ± s.e.m. for each group are shown.

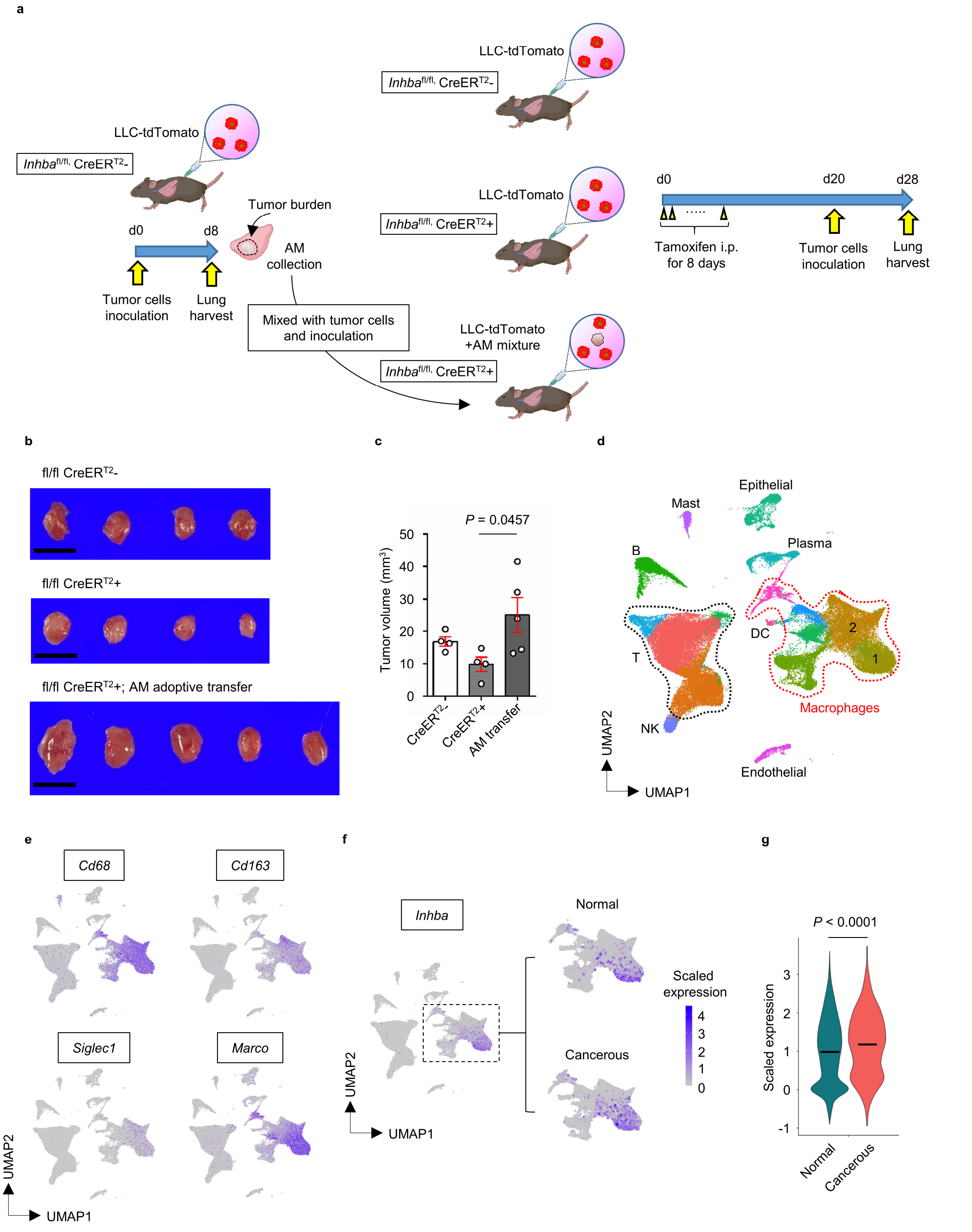

Supplementary Fig. S5. Tumor proliferation is suppressed in INHBA-deficient condition.

a, Experimental design schematic of orthotopic lung cancer model using tamoxifen-inducible *Inhba* knockout mice with or without adoptive transfer of AMs from tumor tissue. **b**, Gross appearance of tumors from *Inhba*$^{fl/fl}$ CreER^{T2}– (upper), *Inhba*$^{fl/fl}$ CreER^{T2}+ without transfer (middle), and *Inhba*$^{fl/fl}$ CreER^{T2}+ with AM transfer (lower) mice. Scale bars; 5 mm. **c**, Tumor volume of *Inhba*$^{fl/fl}$ CreER^{T2}–, *Inhba*$^{fl/fl}$ CreER^{T2}+ without transfer, and *Inhba*$^{fl/fl}$ CreER^{T2}+ with AM transfer mice (n = 4 for *Inhba*$^{fl/fl}$ CreER^{T2}– and *Inhba*$^{fl/fl}$ CreER^{T2}+ without transfer, n = 5 for *Inhba*$^{fl/fl}$ CreER^{T2}+ with AM transfer). Means ± s.e.m. for each group are shown. **d-f**, UMAP plot of single-cell RNA-seq data of CD45$^+$ cells from normal and cancerous human lungs. (**d**) Data from 62,188 cells were analyzed and hierarchically clustered into 20 clusters. (**e**) *Cd68*$^+$, *Siglec1*$^+$, *Cd163*$^+$, and *Marco*$^+$ cell distributions are depicted in the UMAP. (**f**) *Inhba* expression is shown in the UMAP. Normal and cancerous samples are separately represented. **g**, Violin plot showing statistical comparison of *Inhba* expression between normal and cancerous samples. Statistical significance was determined using one-way ANOVA with the Tukey–Kramer post hoc test (**c**) and two-tailed Mann-Whitney *U* test (**g**).

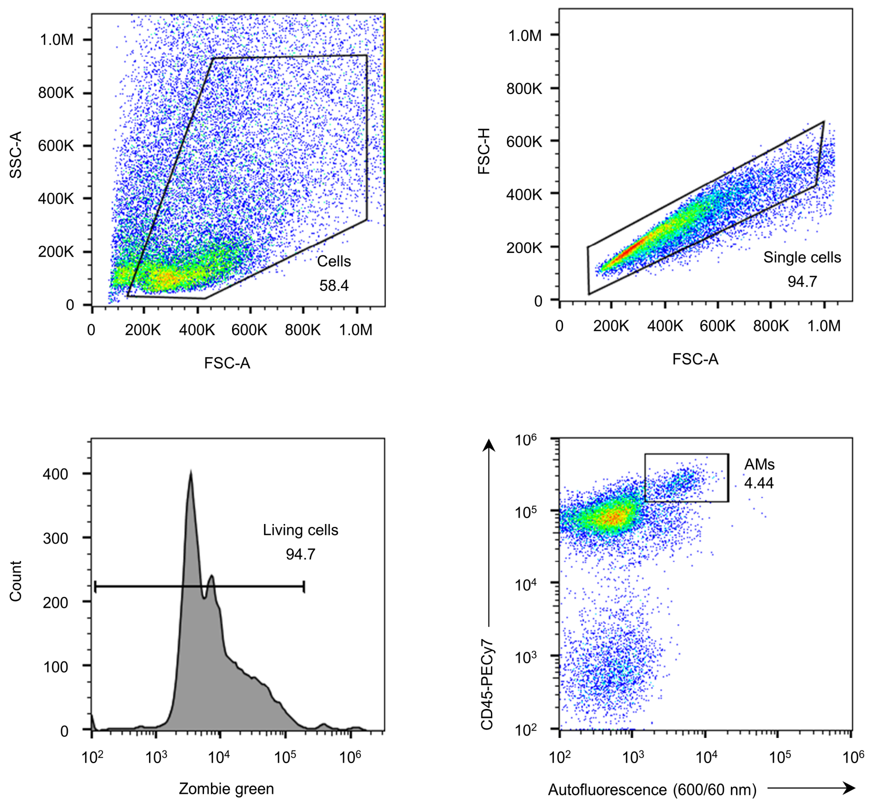

Supplementary Fig. S6. FACS gating strategy.

Gating strategies to evaluate alveolar macrophages in mouse lung.

Supplementary Table 1. Patients information participating in this study

All patients	10
Age (mean)	68 (33-77)
Sex	
Male	4
Female	6
Smoking status	
Never	6
Previous	4
Current	0
Brinkman index (mean)	254 (0-1000)
Histological type	
Adenocarcinoma in situ	5
Invasive adenocarcinoma	5
Tumor size (cm) (mean)	1.83 (0.5-3.0)

Supplementary Table 2. Top 30 genes list by z score with significantly higher expression in clusters 1, 4, and 8 in our scRNA-seq

	names	z scores	logfoldchanges
1	Psap	58.12719	37.907993
2	Ctss	54.142147	32.97345
3	S100a6	54.09692	12.377324
4	H2-K1	51.51078	9.523902
5	Fn1	50.803757	13.069393
6	Btg1	44.736916	8.038326
7	Ctsd	43.70734	37.835403
8	Osm	40.99539	8.095782
9	Cd93	40.29486	2.962887
10	Ccl6	39.61628	36.825386
11	Junb	39.22363	11.449209
12	Ly6e	39.10954	4.219331
13	Pim1	39.093525	5.8839893
14	Syngr1	38.735348	3.8481
15	Cd14	37.526943	4.153883
16	Cd63	37.358265	5.8800063
17	Ier3	36.866646	5.4844666
18	Spp1	36.760548	33.711117
19	Npc2	36.566353	4.2792587
20	Ifi27l2a	36.53933	9.146865
21	Sash1	36.414455	2.8877945
22	B2m	35.28279	9.948016
23	Crip1	34.488342	13.002372
24	H2-D1	33.37018	8.176224
25	Mmp19	32.82604	5.1380067
26	Vps37b	32.76197	3.927517
27	Ecm1	32.40622	3.6515296
28	Gng12	32.39431	2.0754683
29	Trem2	31.84922	2.4744933
30	Hopx	31.425095	3.5770764

Supplementary Table 3. Antibody information used in this study

Molecule	Clone	Conjugate	Vendor	Catalog number	Concentration	Concentration used	Usage
CD45	30-F11	PE-Cyanine7	BioLegend	103114	0.2 mg/mL	1/50	FACS
F4/80	BM8	Brilliant Violet 421	BioLegend	123132	0.2 mg/mL	1/50	FACS
CD11c	N418	APC	BioLegend	117310	0.2 mg/mL	1/50	FACS
CD11b	M1/70	Brilliant Violet 421	BioLegend	101235	0.2 mg/mL	1/50	FACS
Siglec-F	REA798	APC	Miltenyi Biotec	130-112-333	0.15 mg/mL	1/50	FACS
CD11b	REA592	APC	Miltenyi Biotec	130-113-802	0.15 mg/mL	1/50	FACS
MARCO	2359A	None	R&D Systems	MAB29561	0.5 mg/mL	1/500	FACS
Isotype Ctrl	RTK2758	Briliiant Violet 421	BioLegend	400535	0.2 mg/mL	1/50	FACS
Isotype Ctrl	QA16A12	APC	BioLegend	403505	0.2 mg/mL	1/50	FACS
Anti-rabbit	Polyclonal	Alexa Fluor 647	Jackson Immuno Research	711-605-152	1.5 mg/mL	1/200	FACS
CD16/32	2.4G2	None	BD Biosciences	553141	0.5 mg/mL	1/100	FACS
Phospho-SAPK/JNK	Polyclonal	None	Cell Signaling Technology	9251	-	1/1000	Immunoblotting
SAPK/JNK	Polyclonal	None	Cell Signaling Technology	9252	-	1/1000	Immunoblotting
Phospho-p44/42 MAPK (Erk1/2)	D13.14.4E	None	Cell Signaling Technology	4370	-	1/1000	Immunoblotting
p44/42 MAPK (Erk1/2)	137F5	None	Cell Signaling Technology	4695	-	1/1000	Immunoblotting
Phospho-Smad2	138D4	None	Cell Signaling Technology	3108	-	1/1000	Immunoblotting
Smad2	D43B4	None	Cell Signaling Technology	5339	-	1/1000	Immunoblotting
β-Actin	13E5	HRP conjugate	Cell Signaling Technology	5125	-	1/1000	Immunoblotting
Anti-rabbit	-	HRP conjugate	Cell Signaling Technology	7074	-	1/1000	Immunoblotting
Inhibin beta A	polyclonal	None	abcam	ab56057	0.5 mg/mL	1/200	Immunohistochemistry
TTF-1	SP141	None	abcam	ab227652	-	1/100	Immunohistochemistry
CD163	10D6	None	Leica biosystems	CD163	0.049 mg/mL	1/800	Immunohistochemistry
