# Supplementary material for: In vivo induction of activin A-producing alveolar macrophages supports the progression of lung cell carcinoma
Source: Nat Commun. 2023 Jan 17;14:143. doi: 10.1038/s41467-022-35701-8 (PMC9845242; doi:10.1038/s41467-022-35701-8)
Supplement: Supplementary file 2 — Reporting Summary [file 41467_2022_35701_MOESM2_ESM.pdf]

nature portfolio

Corresponding author(s): Masaru Ishii, Takahiro Matsui

Last updated by author(s): Nov 25, 2022

Reporting Summary

Nature Portfolio wishes to improve the reproducibility of the work that we publish. This form provides structure for consistency and transparency in reporting. For further information on Nature Portfolio policies, see our Editorial Policies and the Editorial Policy Checklist.

Statistics

For all statistical analyses, confirm that the following items are present in the figure legend, table legend, main text, or Methods section.

n/a	Confirmed	
[ ]	[x]	The exact sample size (*n*) for each experimental group/condition, given as a discrete number and unit of measurement
[ ]	[x]	A statement on whether measurements were taken from distinct samples or whether the same sample was measured repeatedly
[ ]	[x]	The statistical test(s) used AND whether they are one- or two-sided<br>*Only common tests should be described solely by name; describe more complex techniques in the Methods section.*
[x]	[ ]	A description of all covariates tested
[ ]	[x]	A description of any assumptions or corrections, such as tests of normality and adjustment for multiple comparisons
[ ]	[x]	A full description of the statistical parameters including central tendency (e.g. means) or other basic estimates (e.g. regression coefficient) AND variation (e.g. standard deviation) or associated estimates of uncertainty (e.g. confidence intervals)
[ ]	[x]	For null hypothesis testing, the test statistic (e.g. *F*, *t*, *r*) with confidence intervals, effect sizes, degrees of freedom and *P* value noted<br>*Give P values as exact values whenever suitable.*
[x]	[ ]	For Bayesian analysis, information on the choice of priors and Markov chain Monte Carlo settings
[x]	[ ]	For hierarchical and complex designs, identification of the appropriate level for tests and full reporting of outcomes
[x]	[ ]	Estimates of effect sizes (e.g. Cohen's *d*, Pearson's *r*), indicating how they were calculated

Our web collection on statistics for biologists contains articles on many of the points above.

Software and code

Policy information about availability of computer code

Data collection	The data were collected using TC10 Automated Cell Counter, Illumina HiSeq2500 sequencer in the 75-base single-end mode, Illumina NovaSeq 6000, Thermal Cycler Dice Real-Time System, SH800 cell sorter, SH-9000Lab, Dako Autostainer Link 48, NIS Elements v.4.50.00.
Data analysis	The data were analyzed using FlowJo v. 10, Integrated Differential Expression and Pathway Analysis (iDEP) v. 0.81, Ingenuity Pathway Analysis software (IPA Summer 2022), GraphPad Prism v. 7, Cellranger 5.0.0, Velocyto v0.17.17, Scanpy 1.9.1, ScVelo 0.2.4 (python 3.10.6), ImageQuant LAS-4000 mini system (version 1.3), WES 004-600 (version 3.1.8), ImageJ 1.53t, HALO v3.5.3577, R (version 4.1.2), Seurat (version 4.1.1)

For manuscripts utilizing custom algorithms or software that are central to the research but not yet described in published literature, software must be made available to editors and reviewers. We strongly encourage code deposition in a community repository (e.g. GitHub). See the Nature Portfolio guidelines for submitting code & software for further information.

Data

Policy information about availability of data

All manuscripts must include a data availability statement. This statement should provide the following information, where applicable:

- Accession codes, unique identifiers, or web links for publicly available datasets
- A description of any restrictions on data availability
- For clinical datasets or third party data, please ensure that the statement adheres to our policy

Access to raw RNA-seq and sing-cell RNA-seq data concerning this study were submitted under Gene Expression Omnibus (GEO) accession number GSE193913 and GSE193914.
To review GEO accession GSE193913:

Go to https://www.ncbi.nlm.nih.gov/geo/query/acc.cgi?acc=GSE193913
To review GEO accession GSE193914:
Go to https://www.ncbi.nlm.nih.gov/geo/query/acc.cgi?acc=GSE193914

Field-specific reporting

Please select the one below that is the best fit for your research. If you are not sure, read the appropriate sections before making your selection.

[x] Life sciences [ ] Behavioural & social sciences [ ] Ecological, evolutionary & environmental sciences

For a reference copy of the document with all sections, see nature.com/documents/nr-reporting-summary-flat.pdf

Life sciences study design

All studies must disclose on these points even when the disclosure is negative.

Sample size	We estimated the required sample sizes by considering variations and means, and sought to reach reliable conclusions using sample sizes that were as small as possible. The cost of experiments, complexity and past experience were also used to determine the sample size.
Data exclusions	No data were excluded.
Replication	Experiments included sufficient sample size to ensure the reproducibility of the findings. Representative data was confirmed at least once with an independent experiment. All attempts at replication were successful.
Randomization	For in vivo studies, mice were randomly assigned to each treatment groups within each genotype. For in vitro studies, conditions were randomly assigned to each experimental condition.
Blinding	All assays were not relevant to blinding because the same author is involved in all experiments and analysis.

Reporting for specific materials, systems and methods

We require information from authors about some types of materials, experimental systems and methods used in many studies. Here, indicate whether each material, system or method listed is relevant to your study. If you are not sure if a list item applies to your research, read the appropriate section before selecting a response.

Materials & experimental systems

n/a	Involved in the study	
[ ]	[x]	Antibodies
[ ]	[x]	Eukaryotic cell lines
[x]	[ ]	Palaeontology and archaeology
[ ]	[x]	Animals and other organisms
[ ]	[x]	Human research participants
[x]	[ ]	Clinical data
[x]	[ ]	Dual use research of concern

Methods

n/a	Involved in the study	
[x]	[ ]	ChIP-seq
[ ]	[x]	Flow cytometry
[x]	[ ]	MRI-based neuroimaging

Antibodies

Antibodies used	The antibodies used in this study were as follows: anti-CD45-PECy7 (1:50, clone 30-F11, #103114), anti-F4/80-BV421 (1:50, clone BM8, #123132), anti-CD11c-APC (1:50, clone N418, #117310), and anti-CD11b-BV421 (1:50, clone M1/70, #101235), BV421-rat IgG2a, isotype control (1:50, clone RTK2758, #400535), and APC-human IgG1, isotype control (1:50, clone QA16A12, #403505) were from BioLegend; anti-Siglec-F-APC (1:50, clone REA798, #130-112-333), and anti-CD11b-APC (1:50, clone REA592, #130-113-802) were from Miltenyi Biotec; anti-p-JNK (1:1000, #9251), anti-JNK (1:1000, #9252), anti-p-Erk1/2 (1:1000, #4370), anti-Erk1/2 (1:1000, #4695), anti-p-Smad2 (1:1000, #3108), anti-Smad2 (1:1000, #5339), anti-β-actin (1:1000, #5125), and anti-rabbit IgG, HRP-linked antibody (1:1000, #7074) were from Cell Signaling Technology; anti-Inhibin beta A (1:200, ab56057), and anti-TTF-1 (1:100, clone SP141, ab227652) were from Abcam; anti-CD163 antibody (1:800, clone 10D6) was from Leica biosystems; anti-MARCO antibody (1:500, clone 2359A, MAB29561) was from R&D systems; AlexaFlour647 AffiniPure Donkey anti-Rabbit IgG (1:200, #711-605-152) was from Jackson Immuno Research; and anti-CD16/32 antibody (1:100, clone 2.4G2, #553141) was from BD Biosciences.
Validation	All the antibodies used were commercially available and their validation statements are available on the manufacturer's website described below;<br>anti-CD45-PECy7 (clone 30-F11, #103114, BioLegend)<br>https://www.biolegend.com/ja-jp/search-results/pe-cyanine7-anti-mouse-cd45-antibody-1903?pdf=true&displayInline=true&leftRightMargin=15&topBottomMargin=15&filename=PE/Cyanine7%20anti-mouse%20CD45%20Antibody.pdf

anti-F4/80-BV421 (clone BM8, #123132, BioLegend)
https://www.biolegend.com/ja-jp/products/brilliant-violet-421-anti-mouse-f4-80-antibody-7199?pdf=true&displayInline=true&leftRightMargin=15&topBottomMargin=15&filename=Brilliant%20Violet%20421™%20anti-mouse%20F4/80%20Antibody.pdf

anti-CD11c-APC (clone N418, #117310, BioLegend)
https://www.biolegend.com/ja-jp/products/apc-anti-mouse-cd11c-antibody-1813?pdf=true&displayInline=true&leftRightMargin=15&topBottomMargin=15&filename=APC%20anti-mouse%20CD11c%20Antibody.pdf

anti-CD11b-BV421 (clone M1/70, #101235, BioLegend)
https://www.biolegend.com/ja-jp/products/brilliant-violet-421-anti-mouse-human-cd11b-antibody-7163?pdf=true&displayInline=true&leftRightMargin=15&topBottomMargin=15&filename=Brilliant%20Violet%20421%E2%84%A2%20anti-mouse/human%20CD11b%20Antibody.pdf

BV421-rat IgG2a, isotype control(clone RTK2758, #400535, BioLegend)
https://d1spbj2x7qk4bg.cloudfront.net/ja-jp/products/brilliant-violet-421-rat-igg2a-kappa-isotype-ctrl-7135?pdf=true&displayInline=true&leftRightMargin=15&topBottomMargin=15&filename=Brilliant%20Violet%20421%E2%84%A2%20Rat%20IgG2a,%20%CE%BA%20Isotype%20Ctrl%20Antibody.pdf&v=20221115073101

APC-human IgG1, isotype control (clone QA16A12, #403505, BIolegend)
https://d1spbj2x7qk4bg.cloudfront.net/ja-jp/products/apc-human-igg1-isotype-control-recombinant-antibody-15053?pdf=true&displayInline=true&leftRightMargin=15&topBottomMargin=15&filename=APC%20Human%20IgG1%20Isotype%20Control%20Recombinant%20Antibody.pdf&v=20220401054422

anti-Siglec-F-APC (clone REA798, #130-112-333, Miltenyi Biotec)
https://www.miltenyibiotec.com/JP-en/products/siglec-f-antibody-anti-mouse-reafinity-rea798.html#apc:30-ug-in-200-ul

anti-CD11b-APC (clone REA592, #130-113-802, Miltenyi Biotec)
https://www.miltenyibiotec.com/JP-en/products/cd11b-antibody-anti-mouse-reafinity-rea592.html?utm_source=3rd_labome&utm_medium=product_listing&utm_campaign=4_Recombinant_antibodies_for_smarter_analysis#apc:30-ug-in-200-ul

anti-p-JNK (#9251, Cell Signaling Technology)
https://www.cellsignal.jp/datasheet.jsp?productId=9251&images=1&size=A4

anti-JNK (#9252, Cell Signaling Technology)
https://www.cellsignal.jp/datasheet.jsp?productId=9252&images=1&size=A4

anti-p-Erk1/2 (#4370, Cell Signaling Technology)
https://www.cellsignal.jp/datasheet.jsp?productId=4370&images=1&size=A4

anti-Erk1/2 (#4695, Cell Signaling Technology)
https://www.cellsignal.jp/datasheet.jsp?productId=4695&images=1&size=A4

anti-p-Smad2 (#3108, Cell Signaling Technology)
https://www.cellsignal.jp/datasheet.jsp?productId=3108&images=1&size=A4

anti-Smad2 (#5339, Cell Signaling Technology)
https://www.cellsignal.jp/datasheet.jsp?productId=5339&images=1&size=A4

anti-β-actin (#5125, Cell Signaling Technology)
https://www.cellsignal.jp/datasheet.jsp?productId=5125&images=1&size=A4

anti-rabbit IgG, HRP-linked antibody (#7074, Cell Signaling Technology)
https://www.cellsignal.jp/datasheet.jsp?productId=7074&images=1&size=A4

anti-Inhibin beta A (ab56057, Abcam)
https://www.abcam.co.jp/inhibin-beta-a-antibody-ab56057.pdf

anti-TTF-1 (clone SP141, ab227652, Abcam)
https://www.abcam.co.jp/ttf1-antibody-sp141-ab227652.pdf

anti-CD163 antibody (clone 10D6, Leica bisystems)
https://shop.leicabiosystems.com/ja-jp/ihc-ish/ihc-primary-antibodies/pid-cd163

anti-MARCO antibody (clone 2359A, MAB29561, R&D systems)
https://resources.rndsystems.com/pdfs/datasheets/mab29561.pdf

AlexaFlour647 AffiniPure Donkey anti-Rabbit IgG (#711-605-152, Jackson Immuno Research)
https://www.jacksonimmuno.com/catalog/products/711-605-152

anti-CD16/32 antibody (clone 2.4G2, #553141, BD Biosciences)
https://www.bdbiosciences.com/ds/pm/tds/553141.pdf

Eukaryotic cell lines

Policy information about cell lines

Cell line source(s)	Lewis lung carcinoma (CRL-1642), KLN205 (CRL-1453), AMJ2-C11 (CRL-2456), HEK293T (CRL-3216) were purchased from ATCC.
Authentication	Neither of the cell lines used were authenticated.
Mycoplasma contamination	Not tested
Commonly misidentified lines (See ICLAC register)	No commonly misidentified lines were used in this study.

Animals and other organisms

Policy information about studies involving animals; ARRIVE guidelines recommended for reporting animal research

Laboratory animals	Wild type C57BL/6 and DBA/2 mice were obtained from CLEA Japan. Csf2 knock out (B6.129S-Csf2tm1Mlg/J) mice were obtained from The Jackson Laboratory. MyD88 knock out (B6.129-Myd88tm1Aki/Obs) mice were obtained from Oriental Bio Service. INHBA LoxP/LoxP (fl/fl) mice, provided by Prof. N. Emoto (Kobe pharmaceutical University), were bred with Rosa26CreERT2 mice. For most of the studies, 8-14 week old female mice were used.
Wild animals	No wild animals were used in this study.
Field-collected samples	No field-collected samples were used in this study.
Ethics oversight	Animal experiments were performed in accordance with the experimental animal guidelines of Osaka University under approved protocols.

Note that full information on the approval of the study protocol must also be provided in the manuscript.

Human research participants

Policy information about studies involving human research participants

Population characteristics	This study involves lung tissue from the patient with lung cancer who underwent surgical resection at Osaka University Hospital in 2020. Average age of these patients was 68 years old. 6 out of 10 patients were female and never smoker. 5 out of 10 patients were pathologically diagnosed with invasive adenocarcinoma. Average tumor size of these patients was 1.83 cm.
Recruitment	Lung cancer tissue or normal lung tissue far from cancer lesion were obtained from the patients who underwent surgical resection at Osaka University Hospital in 2020. Selection of patients was random.
Ethics oversight	Research involving human subjects was approved by the Institutional Review Board of Osaka University on June 14, 2019 (Approval No. 18518). Need for individual consent was waived, as this was a retrospective analysis and data were accessed after masking patients' identity.

Note that full information on the approval of the study protocol must also be provided in the manuscript.

Flow Cytometry

Plots

Confirm that:

- [x] The axis labels state the marker and fluorochrome used (e.g. CD4-FITC).
- [x] The axis scales are clearly visible. Include numbers along axes only for bottom left plot of group (a 'group' is an analysis of identical markers).
- [x] All plots are contour plots with outliers or pseudocolor plots.
- [x] A numerical value for number of cells or percentage (with statistics) is provided.

Methodology

Sample preparation	Blood sample was collected from the abdominal vena cava after euthanasia. The lungs from female adult mice were harvested immediately without perfusion and stored in 1× PBS in 1.5mL safe-lock tubes on ice. The lung tissues were minced with autoclaved scissors for about two minutes, and digested with 1 mg/mL collagenase type IV and 3mg/mL dispase in HBSS at 37 °C for 45 minutes. For the coculture analysis with DAMPs, primary AMs were collected from wild type C57BL/6 mouse

	bronchoalveolar lavage fluid. For preparation of DAMPs, LLC cells were suspended and suspernatant was collected via centrifugation after being subjected to five freeze-thaw cycles. Bone marrow samples were harvested from femur and tibia bones and crushed in fluorescence-activated cell sorting (FACS) buffer (1× PBS, 4% FBS, and 2mM EDTA). Disaggregated tissue elements were passed through a 70-µm cell strainer and centrifuged at 300 × g for 5 min to prepare single cell suspensions. Zombie Green (#423111, BioLegend) was used to remove dead cells. Blood and isolated cells were blocked with anti-CD16/32 antibody for 15 min, followed by staining with the antibodies described above for 30 min.
Instrument	SH800 cell sorter (Sony)
Software	FlowJo v. 10
Cell population abundance	After sorting CD45+autofluorescence+F4/80+SiglecF+ cell populations, the sorted cells were passed through the cell sorter machine in order to determine the percentage of positive cells. The percentage of those populations was more than 80 %.
Gating strategy	Preliminary gates were set to exclude cell debris and RBC on FSC/SSC plots. Then single cells were gated on the FSC-H/FSC-A plots. Gating was determined by blank and single cell color-staining. The gated cells were then gated for each desired positive or negative cells according to each determination.

☒ Tick this box to confirm that a figure exemplifying the gating strategy is provided in the Supplementary Information.
